# Absence of diagonal force constants in cubic Coulomb crystals

**DOI:** 10.1098/rspa.2020.0518

**Published:** 2020-12-23

**Authors:** Bartholomew Andrews, Gareth Conduit

**Affiliations:** 1 TCM Group, Cavendish Laboratory, University of Cambridge, Cambridge CB3 0HE, UK; 2 Department of Physics, University of Zurich, Winterthurerstrasse 190, 8057 Zurich, Switzerland

**Keywords:** crystal structure, crystal stability, Coulomb crystal, force constants, density tight-binding model, density nearly-free electron model

## Abstract

The quasi-harmonic model proposes that a crystal can be modelled as atoms connected by springs. We demonstrate how this viewpoint can be misleading: a simple application of Gauss’s law shows that the ion–ion potential for a cubic Coulomb system can have no diagonal harmonic contribution and so cannot necessarily be modelled by springs. We investigate the repercussions of this observation by examining three illustrative regimes: the bare ionic, density tight-binding and density nearly-free electron models. For the bare ionic model, we demonstrate the zero elements in the force constants matrix and explain this phenomenon as a natural consequence of Poisson’s law. In the density tight-binding model, we confirm that the inclusion of localized electrons stabilizes all major crystal structures at harmonic order and we construct a phase diagram of preferred structures with respect to core and valence electron radii. In the density nearly-free electron model, we verify that the inclusion of delocalized electrons, in the form of a background jellium, is enough to counterbalance the diagonal force constants matrix from the ion–ion potential in all cases and we show that a first-order perturbation to the jellium does not have a destabilizing effect. We discuss our results in connection to Wigner crystals in condensed matter, Yukawa crystals in plasma physics, as well as the elemental solids.

## 1. Introduction

The classical theory of crystal stability was extensively studied by Born in the first half of the twentieth century [1]. This seminal work focused on deriving the Born stability criteria based on the elasticity constants, as well as determining the scope of the Cauchy–Born rule of crystal deformation [2]. Since this time, the topic of crystal stability has been revisited from numerous perspectives [3]: from the historic models of ionic matter by Born–Landé [4], Born–Mayer [5] and Kapustinskii [6]; the Hume-Rothery rules for metal alloys [7]; through to sophisticated quantum Monte Carlo simulations in current research [8,9]. However, these works are based on quadratic modes, which we demonstrate can be absent from the most basic ion–ion interaction of many common crystal structures. This motivates us to revisit the stability analysis of prototypical models, which are currently of interest to the electronic structure [3], plasma physics [10] and astrophysics [11] communities.

In this paper, we study the force constants matrix with respect to atomic positions for infinite crystals in the bare ionic, density tight-binding and density nearly-free electron regimes.^1^ Having observed that the ion–ion potential for cubic crystal structures can have no diagonal harmonic contribution, we seek to answer the question of what repercussions this has on preferred crystal structure. By looking at a variety of crystal lattices, motivated by the elemental solids in the periodic table, we draw comparisons between specific structures. We stabilize the ionic crystal for all structures through the inclusion of electrons in our model, we study the stability transition and we use our framework to unify complementary models in the literature. We show that, using this simple yet overlooked observation, insight is gained into low-energy crystal structure relaxation.

We first introduce the underlying theory in §2. We then proceed to examine the bare ionic crystal, and subsequently the density tight-binding and density nearly-free electron regimes in §§3, 4 and 5, respectively. Finally, we summarize the conclusions and implications of the results in §6.

## 2. Theory

We consider an infinite crystal of atoms in three dimensions and at zero temperature.

The general Hamiltonian of the system is

$$\hat{H}={\hat{T}}_{\text{i}}+{\hat{T}}_{\text{e}}+{\hat{V}}_{\text{i-i}}+{\hat{V}}_{\text{e-i}}+{\hat{V}}_{\text{e-e}},$$

where ${\hat{T}}_{\text{i}}$, ${\hat{T}}_{\text{e}}$ are the ion and electron kinetic energies, and ${\hat{V}}_{\text{i-i}}$, ${\hat{V}}_{\text{e-i}}$ and ${\hat{V}}_{\text{e-e}}$ are the ion–ion, electron–ion and electron–electron contributions to the potential energy, respectively.

We work in the Born–Oppenheimer (BO) approximation, where the ions are assumed to be significantly more massive than the electrons and therefore move on much longer time scales. In this approximation, the complete many-body problem may be solved in two steps: first, with the BO Hamiltonian containing only the electronic degrees of freedom and ions assumed fixed in space; and second, with the ions free to move in the previously calculated BO potential energy surface to account for the nuclear contribution to the kinetic energy. For the computation of the inter-atomic force constants in this paper, we use the BO potential energy surface, *E*^BO^ [12]. In the cases where we need the total energy, *E*, we then solve the nuclear problem that includes the kinetic energy of the ions.

The contribution from the electronic kinetic energy is discussed in the sections for the models. The ion–ion, electron–ion and electron–electron potential energies are given by the Coulomb interaction.

Each unit cell of the crystal has an atom at the origin of the cell with position **R**_*I*_ (upper case). There may also be additional atoms in the unit cell with displacement vectors **r**_*i*_ (lower case) relative to **R**_*I*_. The general position of an atom at equilibrium may be written as $R_{Ii}^{0}=R_{I}+r_{i}$. We consider an instantaneous small and finite displacement **u**_*Ii*_ of an atom in the crystal, such that the general position of an atom is given as **R**_*Ii*_ = **R**_*I*_ + **r**_*i*_ + **u**_*Ii*_.

Harmonic lattice dynamics is based on a Taylor expansion of the total energy about structural equilibrium. In the BO approximation, this yields

$$E^{\text{BO}}(\{R_{Ii}\})=E^{\text{BO}}(\{R_{Ii}^{0}\})+\frac{1}{2}\sum_{Ii\alpha ,j\beta }\Phi_{Ii\alpha ,0j\beta }u_{Ii\alpha }u_{0j\beta },$$

where *α*, *β* are Cartesian directions and the adiabatic and harmonic approximations are assumed [12]. The quantity *Φ*_*Iiα*,0*jβ*_ is known as the matrix of force constants, given as

$$\Phi_{Ii\alpha ,0j\beta }={\frac{\partial^{2}E^{\text{BO}}}{\partial u_{Ii\alpha }\partial u_{0j\beta }}|}_{u=0},$$

where *J* = 0 owing to translational invariance. This quantifies the stability of a crystal due to the movement of particular atoms. In periodic solids, it is common to subsequently examine the mass-reduced Fourier transform of the force constants matrix, known as the dynamical matrix, given as

2.1 $$D_{i\alpha ,j\beta }(k)=\frac{1}{\sqrt{m_{i}m_{j}}}\sum_{I}\Phi_{Ii\alpha ,0j\beta }e^{-ik\cdot R_{I}},$$

where *m*_*i*_ is the mass of particle *i* and **k** is the linear momentum vector. The eigenvalues of the dynamical matrix are the squared frequencies, *ω*^2^. The dynamical matrix is used to compute eigenmodes and definitively quantify whether a system is stable.

In 1904 Drude proposed the paradigmatic model of a crystalline solid to be atoms connected by springs, which implies that the atoms move in a harmonic potential [13–15]. Here we focus on a crucial contribution to this atom–atom potential, the ion–ion interaction, and show that the ions in cubic crystals are not necessarily bound by a harmonic potential. To demonstrate this statement, we assume a one-component ionic lattice in the absence of any background charge and analyse the components of the matrix of force constants in turn.

First, we examine the diagonal matrix of force constants with respect to the motion of a single ion, *Φ*_0*iα*,0*iβ*_. Since the second derivative is with respect to the position of a single ion, *Φ*_0*iα*,0*iβ*_ = 0 for *α* ≠ *β* by symmetry. Furthermore, the sum over Cartesian directions for this matrix of force constants is equivalent to the Laplacian of the BO energy, such that $\sum_{\alpha }\Phi_{0i\alpha ,0i\alpha }=\nabla_{0i}^{2}E^{\text{BO}}$. Hence, the Poisson equation of Gauss’s theorem demands $\sum_{\alpha }\Phi_{0i\alpha ,0i\alpha }=0$, which is true for all crystal structures. For cubic crystals, symmetry implies *Φ*_0*iα*,0*iα*_ = 0 and therefore the ions are not harmonically bound; for non-cubic crystals, $\sum_{\alpha }\Phi_{0i\alpha ,0i\alpha }=0$ implies that some terms will be positive and other terms will be negative.

Second, we examine the matrix of force constants, *Φ*_*Iiα*,0*iβ*_, with respect to the motion of two ions that reside in different unit cells, one in cell 0 and the other in cell *I*.^2^ In this case, the second derivative is mixed and so the sum over Cartesian directions no longer corresponds to the Laplacian. After perturbing the ion in unit cell 0, we subsequently need to perturb the corresponding ion in unit cell *I* to obtain the cross terms. We demonstrate in electronic supplementary material, section SI that for all crystal structures we obtain the result $\sum_{\alpha }\Phi_{Ii\alpha ,0i\alpha }=0$. We note that this two-ion result with both ions moving parallel to each other has a pleasing analogy to the Poisson equation for the motion of a single ion. In electronic supplementary material, section SI, we also consider the non-parallel motion of the two ions, which for centrosymmetric crystals yields the corollary $\sum_{I\neq 0}\Phi_{Ii\alpha ,0i\beta }=0$, where the summation is over unit cells *I*. To include the *I* = 0 term in the summation, which corresponds to the motion of a single ion, we can use the result from the previous paragraph that *Φ*_0*iα*,0*iβ*_ = 0 for cubic crystals. This implies that the full sum $\sum_{I}\Phi_{Ii\alpha ,0i\beta }=0$ holds for all centrosymmetric cubic crystals, which includes all of the cubic space groups considered in this paper.

Using the above analysis, we have shown that $\sum_{\alpha }\Phi_{Ii\alpha ,0i\alpha }=0$ for all crystal structures and $\sum_{I}\Phi_{Ii\alpha ,0i\beta }=0$ for all centrosymmetric cubic crystal structures. Substituting the latter result into equation (2.1), we see that there is at least one momentum mode where the diagonal dynamical matrix with respect to ion positions, *D*_*iα*,*iβ*_, is identically zero. Since the trace of the dynamical matrix is equal to the sum of its eigenvalues, *D*_*iα*,*iβ*_ = 0 implies that centrosymmetric cubic crystals are neither stabilized nor destabilized by a harmonic term. For other crystals, we note that the trace of the diagonal dynamical matrix is zero, $\sum_{\alpha }D_{i\alpha ,i\alpha }=0$. Therefore, if some modes are stable (*ω*^2^ > 0) others will be necessarily unstable (*ω*^2^ < 0). These results provide strong motivation to revisit the stability of crystals.

In this paper, we study the diagonal matrix of force constants, ***Φ***_*I*_ ≡ *Φ*_*Iiα*,0*iβ*_. We focus on the diagonal (*i* = *j*) elements of the matrix of force constants, since they are sufficient to demonstrate that a system is *not* stable (see electronic supplementary material, section SII).^3^ Moreover, in cases where ***Φ***_*I*_ = **0**, we additionally examine the symmetry-contracted fourth-order diagonal force constant matrix, **X**_*i*_ ≡ *X*_*Iiα*,0*iβ*_ (see electronic supplementary material, section SIII).^4^

With the strategy and motivation in place, we still face the challenge of calculating the energy. We therefore turn to three limits where we can make progress: the bare ionic crystal, and subsequently the density tight-binding and density nearly-free electron models.

## 3. Bare ionic model

We start with the simplest system that demonstrates the concept of this paper. For the bare ionic crystal, we consider a one-component crystal of Coulomb point charges of equal sign and infinite extent. This is typical of the systems studied in plasma physics [16], albeit without a background of positive charges. Working in atomic units, the Coulomb potential is *V*(**R**) = |**R**|^−1^, corresponding to repulsive interactions between the point charges. Our strategy is to demonstrate that cubic crystals can have a zero matrix of force constants with respect to the motion of a single ion, and therefore centrosymmetric cubic crystals are not necessarily stabilized or destabilized at harmonic order.

In §3a, we discuss the background and key developments in the field of Coulomb crystals, and in §3b we analyse our numerical results.

### (a) Background

Coulomb crystals are defined by the dominant role of the Coulomb interaction and the simple form of their constituents [17]. In this paper, we consider a special type of ‘transient Coulomb’ crystal, categorized as an unconfined and infinite, one-component system with repulsive interactions. However, the study of Coulomb crystals extends beyond this limiting case and has a history spanning over a century [18].

The earliest study of a one-component system was by Madelung in 1918 [18], where he showed that an infinite array of point charges can form an ordered state. Two decades later, Wigner predicted, in his seminal paper, that the electron jellium in metals can form a body-centred cubic crystal at sufficiently low densities [19,20]. The subsequent numerical and experimental confirmation of Wigner crystals sparked interest in the condensed matter community, and a plethora of papers on the general theory [21–27] and stability [28–31] of these systems followed, including detailed quantum Monte Carlo simulations [32–39]. From the plasma physics perspective on the other hand, interest in strongly coupled plasmas, i.e. plasmas where the average Coulomb energy of a particle is much greater than its average kinetic energy [40], led to the prediction that three-dimensional, one-component Coulomb plasmas can also form a body-centred cubic crystal at sufficiently *high* densities and/or low temperatures [41]. It was subsequently realized that these two conclusions could be reconciled as opposite density limits of the same problem.^5^ All of these models, however, include a homogeneous positive background of charges to stabilize the system. Indeed, there are two ways to stabilize a repulsive Coulomb crystal: a homogeneous oppositely charged background or confinement [17].

Work on confined plasmas has been performed in a variety of contexts [40]. Most notably, the structure and Madelung energy [42] as well as the melting of ordered states [43] in spherical Coulomb crystals have been studied in the last 30 years. These systems can also be probed and manipulated experimentally using ions confined to Penning [16] or Paul [44] traps, with motivation provided by the recent discovery of crystalline plasmas of dust particles in astrophysics [11] as well as the industrial success of quantum dot technology [45]. For all of these confined systems, however, the resulting crystal structure is strongly dependent on the shape of the trap [43]. Therefore, no general statements can be made about the equilibrium structure.

In this section, we study the instability of unconfined Coulomb crystals, which we stabilize in later sections through the inclusion of an oppositely charged background.

### (b) Analysis

We first calculate the BO energy of a lattice of ions. We examine the Bravais lattices: simple cubic (cub), body-centred cubic (bcc) and face-centred cubic (fcc). Additionally, we study the diamond (dia) lattice structure, from the fcc family, separately, as it is of special interest owing to its extreme material properties, such as hardness and thermal conductivity. We also include the hexagonal close-packed (hcp) and double hexagonal close-packed (dhcp) structures in our analysis, from the hexagonal Bravais lattice family, because of their ubiquity in nature (see electronic supplementary material, section SIV).

In order to perform the summation over lattice sites in this section we use a rotationally symmetric summation scheme. We start by defining all unit cells with an atom at the origin and then incrementally add atoms in concentric shells. The long-range contribution is incorporated using the classical Ewald method [46] and we compute this summation until convergence to the desired precision. The full details of the numerical model are discussed in electronic supplementary material, section SV.

**Figure 1.**
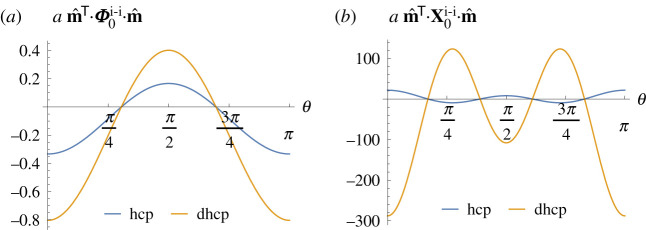
Angular variation in the inner product of the matrices of force constants with a direction vector **m** at *ϕ* = *π*/2 and unit radius, in units of *a*^−1^. Plots are shown for the (*a*) second-order and (*b*) fourth-order terms for the hcp and dhcp crystal structures. Note that the inner product of the diagonal matrices of force constants is azimuthally symmetric for these systems. The minimizing directions are recorded in tables 1 and 2. (Online version in colour.)

The diagonal matrices of force constants, directions of greatest instability and minimal eigenvalues for these crystals are shown in table 1. For all crystal structures, the trace of the diagonal matrix of force constants is zero. For cubic systems (cub, bcc, fcc, dia) the diagonal harmonic term is identically zero, whereas for hexagonal systems (hcp, dhcp) the diagonal force constant matrices are indefinite, which implies the system is at a saddle point. We also see that hexagonal structures are stable to diagonal perturbations in the *xy*-plane, but most unstable to diagonal perturbations in the *z*-direction, as illustrated in figure 1*a*. The dhcp system is more unstable than the hcp system with this metric because of the higher density of ions.

**Table 1.** Diagonal matrices of force constants and minimizing directions for the ion–ion interaction expansion about equilibrium, at second order with lattice spacing, *a*. The cubic crystals are denoted by *C* ∈ {cub, bcc, fcc, dia} and the hexagonal crystals by *H* ∈ {hcp, dhcp}. ***Φ***_0_ is the Hessian; ${\hat{\text{m}}}_{2}$ is the normalized eigenvector corresponding to the lowest eigenvalue of the Hessian; and ${\hat{\text{m}}}_{2}^{⊺}\cdot \Phi_{0}\cdot {\hat{\text{m}}}_{2}$ is the projection of the Hessian in the minimizing direction. All values are given to the precision up to which they have converged, or three significant figures, whichever is lower.

crystal	*a****Φ***^i−*i*^_0_	${}_{\text{i-i}}{\hat{\text{m}}}_{2}$	${}_{\text{i-i}}{\hat{\text{m}}}_{2}^{⊺}\cdot (a\Phi_{0}^{\text{i-i}})\cdot {}_{\text{i-i}}{\hat{\text{m}}}_{2}$
*C*	**0**	−	0
*H*	$-\frac{k}{2}\left(\begin{array}{ccc} 1 & 0 & 0\\ 0 & 1 & 0\\ 0 & 0 & -2 \end{array}\right)$	$\pm {\hat{\text{e}}}_{z}$	$\begin{array}{rl} k_{\text{hcp}} & =-0.33\\ k_{\text{dhcp}} & =-0.8 \end{array}$

**Figure 2.**
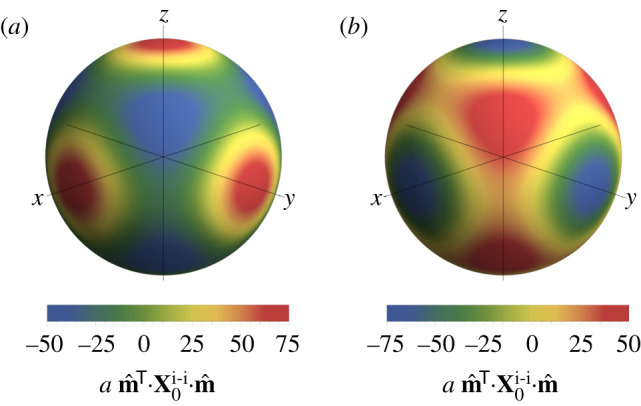
Angular variation in the inner product of the fourth-order diagonal matrices of force constants with a direction vector **m** at unit radius, in units of *a*^−1^. Plots are shown for the (*a*) cub and (*b*) bcc crystal structures. The plots for the fcc and dia crystal structures have an identical form to (*b*) with different scales. The scales and minimizing directions are recorded in table 2. (Online version in colour.)

Having found that the cubic crystal stability test can be inconclusive at second order, we turn to a higher order expansion. An analogous table for the fourth-order diagonal matrices of force constants is shown in table 2.^6^ At this order, the cubic systems do not have vanishing contributions; instead, they demonstrate a fourth-order instability. Note that the form of the fourth-order matrices is similar in each case, with a varying pre-factor. Plots of the angular variation of these fourth-order matrices are shown in figure 2. As for the hexagonal systems at second order, the system is again at a saddle point. In this case, the configuration is stable to perturbations in the Cartesian basis directions for cub; and in the diagonal directions for bcc, fcc and dia crystals, and vice versa. For completeness, we show that the fourth-order matrices for the hexagonal systems are also indefinite, as shown in figure 1*b*. In the dhcp case, the minimizing directions are again $\pm {\hat{e}}_{\text{z}}$, whereas for the hcp system the most unstable directions have now shifted to *θ* = 0.857, *π* − 0.857. The angular variation for the hexagonal systems is rotationally symmetric about the *z*-axis, since the *x*- and *y*-eigenvalues are the same. Note that since higher order (in)stabilities are always weaker than lower orders, it is unnecessary to examine the higher order terms for these hexagonal systems. As seen for the second-order case, the magnitude of the instabilities is determined by the ion density.

**Table 2.** Diagonal force constant matrices and minimizing directions for the ion–ion interaction expansion about equilibrium, at fourth order, with the same conventions as table 1. Fourth-order matrices are symmetry contracted as described in electronic supplementary material, section SIII.

crystal	*a***X**^i^−*i*_0_	${}_{\text{i-i}}{\hat{\text{m}}}_{4}$	${{}_{\text{i-i}}{\hat{\text{m}}}_{4}^{\circ 2}}^{⊺}\cdot (a{\text{X}}_{0}^{\text{i-i}})\cdot {}_{\text{i-i}}{\hat{\text{m}}}_{4}^{\circ 2}$
cub	$74.6\left(\begin{array}{ccc} 1 & -1.5 & -1.5\\ -1.5 & 1 & -1.5\\ -1.5 & -1.5 & 1 \end{array}\right)$	$\frac{1}{\sqrt{3}}(\pm {\hat{\text{e}}}_{x}\pm {\hat{\text{e}}}_{y}\pm {\hat{\text{e}}}_{z})$	−49.7
bcc/fcc/dia	$k\left(\begin{array}{ccc} 1 & -1.5 & -1.5\\ -1.5 & 1 & -1.5\\ -1.5 & -1.5 & 1 \end{array}\right)$	$\left\{\pm {\hat{\text{e}}}_{x},\pm {\hat{\text{e}}}_{y},\pm {\hat{\text{e}}}_{z}\right\}$	$\begin{array}{rl} k_{\text{bcc}} & =-74.6\\ k_{\text{fcc}} & =-181\\ k_{\text{dia}} & =-2570 \end{array}$
hcp	$8.1\left(\begin{array}{ccc} 1 & 1 & 0\\ 1 & 1 & 0\\ 0 & 0 & 0 \end{array}\right)+21.6\left(\begin{array}{ccc} 0 & 0 & -1.5\\ 0 & 0 & -1.5\\ -1.5 & -1.5 & 1 \end{array}\right)$	*θ* = 0.857, *π* − 0.857	−9.3
dhcp	$-108\left(\begin{array}{ccc} 1 & 1 & 0\\ 1 & 1 & 0\\ 0 & 0 & 0 \end{array}\right)-288\left(\begin{array}{ccc} 0 & 0 & -1.5\\ 0 & 0 & -1.5\\ -1.5 & -1.5 & 1 \end{array}\right)$	$\pm {\hat{\text{e}}}_{z}$	−288

This lack of a diagonal harmonic contribution to the energy in cubic systems appears to contradict the 1904 quasi-harmonic model for a crystal of atoms connected by springs [13–15]. However, as stated before, it is a natural consequence of Gauss’s theorem (∂_*xx*_ + ∂_*yy*_ + ∂_*zz*_)*E*^BO^ = 0. In a system with cubic symmetry all terms in Gauss’s theorem must be identical so each must be zero, ∂_*αα*_*E*^BO^ = 0. Furthermore, ∂_*αβ*_*E*^BO^ = 0 for these examples by symmetry. Conversely, in systems without cubic symmetry, we can say that if in some direction the second derivative is positive, then in others it must be negative to satisfy Gauss’s theorem, and so will never be stable by geometry. The changing sign in the fourth-order derivative in cubic systems is expected as Gauss’s theorem requires the net electron flux through a closed surface to be zero, so positive contributions must be counterbalanced by negative contributions. We therefore deduce that it is inevitable that crystalline solids are not stabilized at any order by contributions from the ion–ion potential.

Note that in this section we have considered a one-component ionic crystal without a neutralizing background to show that cubic structures have the weakest (fourth-order) instability with respect to the motion of a single ion. In §5 we will show that if a constant neutralizing background is introduced, this would provide a quadratic restoring potential for the ions, which would compensate for this instability. This holds even for non-cubic systems, since it can be shown that the stabilizing contribution to the dynamical matrix from the constant uniform background is greater than the destabilizing contribution from the purely repulsive ionic crystal.

## 4. Density tight-binding model

We found that the bare crystal of ions is not stable and so, motivated by the need to stabilize the system, we now consider the simplest model to include electrons to bind the ions: the density tight-binding model. The electrons are tightly bound to each nucleus with a spherical effective charge density parametrized by core and valence orbital radii.

We start by analysing the model and phase diagram in §4a, and then discuss the interpretation in §4b.

### (a) Analysis

In the density tight-binding approximation, the electrons are situated directly on top of and nearby to the ions. We consider ions that have only spherically symmetric (s-type) orbitals, with the electron density distribution

$$\rho_{\text{E}}(r;c,a_{\text{e}})\propto \frac{1}{1+\exp(2(|r|-c)/a_{\text{e}})},$$

where the normalization factor to give net charge neutrality is given in electronic supplementary material, section SVI A 1. Here **r** denotes the displacement of the electron relative to the origin of its associated ion, and *c*, *a*_e_ characterize the core and valence orbital radii, respectively. The factor of 2 ensures that the associated wave function, defined by *ρ* = |Ψ|^2^, reduces to the hydrogenic atom solution, ∼exp ( − |**r**|/*a*_e_), in the extreme density tight-binding approximation: *c* ≪ *a*_e_ ≪ *a*. We choose this form of the electron orbital density [37] because it is analytically well behaved for the required derivation and has the correct scaling behaviour (see electronic supplementary material, section SVI A 1). Throughout our calculations, we work to leading order in the density tight-binding approximation. In practice, this implies results up to first order in the small core radial parameter (*c*/*a*_e_) and second order in the valence radial parameter (*a*_e_/*a*).

As mentioned in §2, to compute the total energy, *E*, we first solve the electronic problem with ions assumed fixed and then we allow the ions to move in the Born–Oppenheimer potential energy surface to account for the ionic contribution to the kinetic energy.

There are two contributions to the electronic kinetic energy: the energy due to confinement and the energy due to tunnelling. We note that the expectation value of the total electronic kinetic energy due to confinement is effectively independent of atom positions, since each potential well in the vicinity of an ion is approximately the same shape. Moreover, the contribution from the electrons tunnelling into neighbouring wells is exponentially small. Therefore, the expectation value of the total electronic kinetic energy is constant with respect to atom configurations.

We calculate the ion–ion, electron–ion and electron–electron contributions to the potential energy based on the electron orbital ansatz up to the approximations detailed above. We subsequently add on the contribution to the energy due to the Pauli repulsion of the overlapping electron orbitals, evaluated at the optimal effective radius of atoms in a spherical packing. Finally, we relax the crystal structure to find the optimal lattice constant, *a*. We perform the calculation for each of the crystal structures: cub, bcc, fcc, dia, hcp and dhcp.

For both the kinetic and potential energies, we use the same rotationally symmetric summation scheme for the crystal introduced in §3. The details of the numerical model are discussed in electronic supplementary material, section SVI.

**Figure 3.**
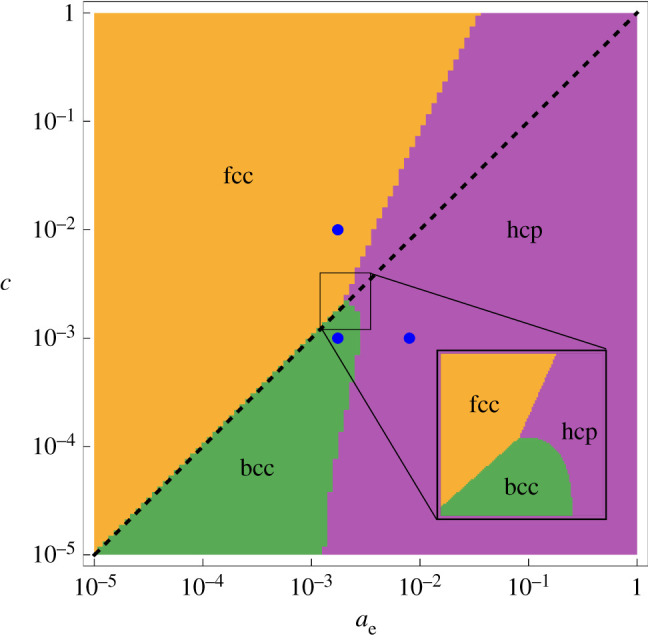
Phase diagram of the lowest energy crystal structure out of {cub, bcc, fcc, dia, hcp, dhcp} at the optimum lattice constant, summed out to eight shells. The black line separates the valid region for the density tight-binding model: the lower right-hand triangle at *c* < *a*_e_. The blue points, {(1.75 × 10^−3^, 10^−2^), (1.75 × 10^−3^, 10^−3^), (8 × 10^−3^, 10^−3^)}, are analysed in figure 4.Inset: Higher resolution plot of the region enclosed by the black square, highlighting the tricritical point. The diagrams are plotted to a resolution of 100^2^ points. (Online version in colour.)

In figure 3, we show the phase diagram of the stable crystal structures with the lowest energy out of the cub, bcc, fcc, dia, hcp and dhcp lattices. We note that all of the crystal structures are stable with respect to their dynamical matrices in this model and so the preferred crystal structure is determined by the total energy hierarchy. Out of the six crystal structures considered, the hcp, fcc and bcc structures are found to be the preferred phases. We present a higher resolution close-up of the tricritical point in the inset of figure 3 to analyse the features of interest. The tricritical point is at (*a*_e_, *c*) = (2.04, 2.13) × 10^−3^ with three transition lines: fcc-bcc at *c* ∝ *a*_e_; fcc-hcp at $c\propto a_{\text{e}}^{2.5}$; and bcc-hcp at *c* = 2.13 × 10^−3^ in the vicinity of the tricritical point. Since all phase transitions between allotropes of crystal structures are first order, the tricritical point is valid with respect to the vertex rule. Note that other than the restriction imposed by the density tight-binding approximation, in this context $c≲a_{\text{e}}$, the phase diagram may be extended in both directions.

**Figure 4.**
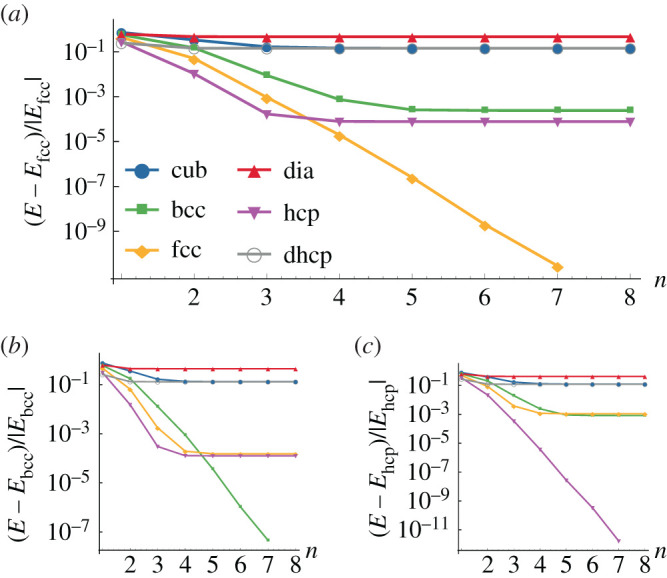
Detailed analysis of the blue points depicted in figure 3, such that (*a*) is at (*a*_e_, *c*) = (1.75 × 10^−3^, 10^−2^), (*b*) is at (1.75 × 10^−3^, 10^−3^) and (*c*) is at (8 × 10^−3^, 10^−3^). The plots show the fractional deviation of the energies from the lowest energy value at eight shells, [*E* − *E*_c_.s.(*n* = 8)]/|*E*_c_.s.(*n* = 8)|, against the number of shells in the summation, *n*. (Online version in colour.)

Now that we have constructed the phase diagram, we verify the convergence of our calculations. Figure 4 shows a detailed analysis of the blue points depicted in figure 3. Most importantly, we see from plots of the total energy against number of shells of ions in the summation that convergence is reached at approximately five shells. Therefore, we plot the phase diagram by summing over eight shells, deep into the converged region. We consistently observe that {bcc, fcc, hcp} forms the energetically favourable subset of crystal structures.

### (b) Discussion

In this section, we progressed from the ionic crystal in §3 by introducing tightly bound electrons to stabilize the system. Three phases emerged with noticeably lower energy: hcp, bcc and fcc. Each is the ground state in different limits, and all have been separately noted before, hence the density tight-binding model presented allows us to reconcile previous findings in a unified framework. We now discuss how these phases emerge in the three separate limits of our density tight-binding model.

It has been known for a long time that three-dimensional Coulomb crystals have a bcc symmetry [41], where the term ‘Coulomb crystal’ in plasma physics refers to strongly coupled charged particles with a neutralizing background [17]. In the density tight-binding limit (*c* ≪ *a*_e_ ≪ *a*), this is effectively equivalent to the system presented in figure 3. The particle interactions are Coulomb-like, since the effect of the well is still minimal, and the presence of the electrons provides the neutralizing background, albeit highly concentrated around the ions. Therefore, it is unsurprising that we see the same bcc ground-state crystal structure. This also has parallels to a Wigner crystal, where the decay of the electronic wave function is sufficiently slow to stabilize the crystal [19].

As soon as we move into the region where *c* > *a*_e_, we modify the effective interaction through screening. In this region we observe the behaviour of screened Coulomb charges, and when *c* ≫ *a*_e_ and *c* ≫ *a* we observe the density nearly-free electron model. Indeed, it has been shown by Hamaguchi *et al.* [47] that three-dimensional Yukawa crystals have a bcc and fcc phase. They show that there exist two solid phases for the Yukawa crystal: bcc at small screening parameter and a transition to fcc when the screening parameter is increased, which corresponds to moving vertically upwards in our phase diagram.

**Figure 5.**
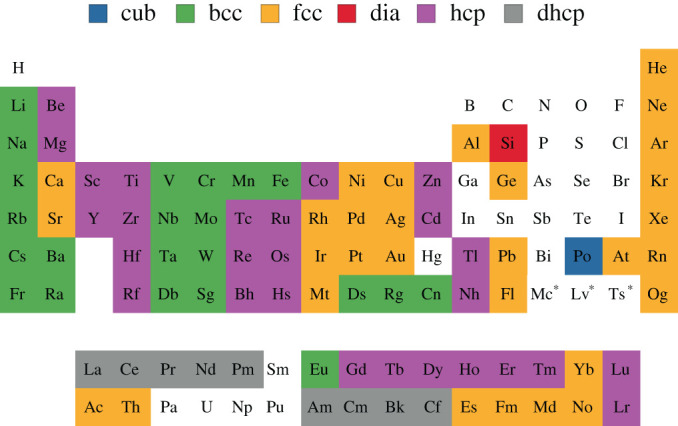
The periodic table of elements labelled according to the crystal structure of their thermodynamically most stable allotrope [49]. The white cells correspond to elements with a crystal structure that is not in the set {cub, bcc, fcc, dia, hcp, dhcp}. The crystal structure of elements marked with an asterisk is not known. (Online version in colour.)

In addition to these extreme limits, our model provides insight into the transition from extreme matter to real materials. From figure 3, we can see that as *a*_e_ is increased, that is, the valence electron radius is increased and the density tight-binding approximation is relaxed, the hcp structure is energetically favourable, for both *c* < *a*_e_ and *c* > *a*_e_. This shows that in many materials the crystal lattice begins to favour high symmetry and a high packing factor. The limit applies to many of the lanthanides and actinides that are in the tight-binding regime [48]. Moreover, as shown in figure 5 and discussed in electronic supplementary material, section SIV, the hexagonal structure is the most common Bravais lattice in the periodic table. More generally, the vast majority of the periodic table is composed of the bcc, fcc and hcp crystal structures;^7^ identified here are the three most energetically favourable structures.

## 5. Density nearly-free electron model

In contrast to the density tight-binding model, where the Bohr radii of the atoms are much smaller than the inter-atomic spacing, we now consider the opposite ‘density nearly-free electron’ limit, where the Bohr radii mostly overlap. This is applicable to a variety of simple metals in the periodic table, and particularly the alkali metals. In the weak-binding or density nearly-free electron model, we perform first-order perturbation theory about the jellium model, where the electron density is uniform. Our strategy is to focus on the ion motions found in §3 to be not governed by a harmonic potential, and then investigate whether the electron cloud can compensate for this. The details of the electron cloud densities in this model are presented in electronic supplementary material, section SVII.

**Figure 6.**
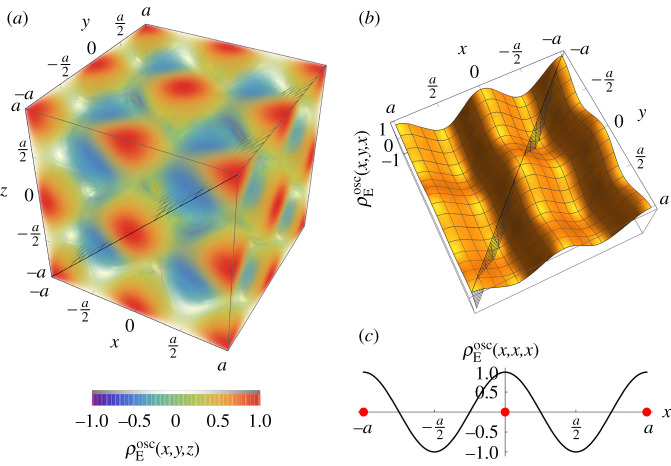
(*a*) Three-, (*b*) two- and (*c*) one-dimensional plots of the oscillatory part of the electron cloud density, $\rho_{\text{E}}^{\text{osc}}(x,y,z)=[\cos(kx)+\cos(ky)+\cos(kz)]/3$ with *k* = 2*π*/*a*, for the simple cubic lattice in the density nearly-free electron model. (*a*) Colour and opacity both denote the magnitude of $\rho_{\text{E}}^{\text{osc}}(x,y,z)$. (*b*) Plot of the meshed cross-section depicted in (*a*), through the density extrema. (*c*) Plot of the meshed cross-section depicted in (*b*), through the density extrema. The red points illustrate the positions of the ions. (Online version in colour.)

The density nearly-free electron model comprises a lattice of ions with Coulomb repulsion, as studied in §3, together with an oscillatory and near-uniform electron cloud density, *ρ*_E_. The expectation value of the total electronic kinetic energy in this model is therefore directly proportional to the Fermi energy. In accordance with first-order perturbation theory, this electron cloud density may be split into two parts, $\rho_{\text{E}}=\rho_{\text{E}}^{\text{cst}}+\rho_{\text{E}}^{\text{osc}}$, with $\rho_{\text{E}}^{\text{cst}}$ corresponding to the constant jellium-like density and $\rho_{\text{E}}^{\text{osc}}$ corresponding to the oscillatory density due to the electron–ion interaction and the geometry of the ionic lattice. An example of the oscillatory electron cloud density for the simple cubic lattice is shown in figure 6. In each case, we ensure that the density range is normalized such that $\max(\rho_{\text{E}}^{\text{osc}})=u$, where *u* is the oscillation strength, and that the integral of $\rho_{\text{E}}^{\text{osc}}$ over a unit cell is equal to zero.

In order to calculate the total diagonal force constant matrix, we proceed by summing the ion–ion, electron–ion and electron–electron contributions from the BO potential. For the ion–ion contribution, we take results directly from §3. Note that, when performing the real-space summation over shells for the ion–ion contribution, the zeroth-order contribution to the BO energy is divergent, whereas the matrix of force constants converges. In fact, there are divergent zeroth-order contributions for the electron–ion and electron–electron contributions too, corresponding to the jellium-like term in the electron density. These divergent terms cancel, which is reflected in the Ewald summation. However, we gain additional insight by directly calculating the diagonal force constants matrix in each case. The constant electron–ion contribution to the diagonal force constants matrix must satisfy $\sum_{\alpha }\Phi_{0i\alpha ,0i\alpha }^{\text{e-i, cst}}=4\pi \rho_{\text{E}}^{\text{cst}}$ by Poisson’s law. The uniform neutralizing background stabilizes any crystal with respect to diagonal force constants and its contribution is summarized in table 3.

**Table 3.** Electron–ion contributions to the diagonal force constants matrix in the density nearly-free electron model. The cubic crystals are denoted by *C* ∈ {cub, bcc, fcc}. The prefactors for the constant electron–ion contributions for cubic systems are {*k*_cub_, *k*_bcc_, *k*_fcc_} = {1, 2, 4}. **I** and $\tilde{u}$ denote the identity matrix and dimensionless oscillation strength, respectively.

crystal	*a*(***Φ***^e^−*i*, *cst*_0_ + ***Φ***^e^−*i*, *osc*_0_)
*C*	$\frac{4\pi }{3}\left(k+\sqrt{2\pi }\tilde{u}\right)\text{I}$
hcp	$\frac{4\sqrt{2}\pi }{41}\left(1+\sqrt{\pi }\tilde{u}\right)\left(\begin{array}{ccc} 16 & 0 & 0\\ 0 & 16 & 0\\ 0 & 0 & 9 \end{array}\right)$
dhcp	$\frac{4\sqrt{2}\pi }{697}\left(2+\sqrt{\pi }\tilde{u}\right)\left(\begin{array}{ccc} 218 & 0 & 0\\ 0 & 218 & 0\\ 0 & 0 & 261 \end{array}\right)$

The oscillatory electron–ion contribution to the BO energy, $E_{\text{e-i}}^{\text{BO, osc}}(u)=-2\sum_{I}\int V_{\text{i}}(R_{I}-u+r_{\text{e}})\rho_{\text{E}}^{\text{osc}}(r_{\text{e}})dr_{\text{e}}$, may be simplified by noting that all ions are equivalent and so we can focus on the ion at the origin. Subsequently calculating the energy per atom allows us to drop the summation over ions and write

5.1 $$E_{\text{e-i}}^{\text{BO, osc}}(u)=-2\int V_{\text{i}}(-u+r_{\text{e}})\rho_{\text{E}}^{\text{osc}}(r_{\text{e}})\;dr_{\text{e}},$$

where *V*_i_ is given by the Coulomb potential and the oscillatory part of the electron density is approximated by a cosine function (see electronic supplementary material, section SVII).

Finally, for the electron–electron contribution, $E_{\text{e-e}}^{\text{BO}}=\sum_{I}\iint V_{\text{e}}(R_{I}-u+r_{\text{e}}-u_{\text{e}})\rho_{\text{E}}(r_{\text{e}})\rho_{\text{E}}(u_{\text{e}})dr_{\text{e}}du_{\text{e}}$, we may drop the summation by the same argument. Note also that the electron potential does not depend on the displacement of the central ion. Hence, excluding the zeroth-order term and working to first order in the perturbation strength, we may write the oscillatory contribution to the electron–electron BO energy as

5.2 $$E_{\text{e-e}}^{\text{BO, osc}}=2\rho_{\text{E}}^{\text{const}}\int_{r_{\text{e}}\in \begin{array}{c} \text{unit}\\ \text{cell} \end{array}}\int_{u_{\text{e}}\in R^{3}}V_{\text{e}}(r_{\text{e}}-u_{\text{e}})\rho_{\text{E}}^{\text{osc}}(u_{\text{e}})\;du_{\text{e}}dr_{\text{e}},$$

where the factor of 2 is from the addition of both cross terms, and *V*_e_ is again given by the Coulomb potential. For all structures the positive and negative regions of the oscillating electron density $\rho_{\text{E}}^{\text{osc}}$ cancel and therefore the oscillatory electron–electron contribution is zero, *E*^BO^, osc_e_−*e* = 0.

The summation of the leading-order terms from §3, the jellium contribution, as well as the oscillatory contributions from equations (5.1) and (5.2) yields the total BO energy, the diagonal matrix of force constants, and hence an instability discriminant.

The harmonic energy contributions for the crystal structures is summarized in table 3. We note that all of the electron–ion contributions are positive at this order. We can see that the cubic structures all have isotropic matrices, whereas the hexagonal structures are only isotropic in the *xy*-plane, as expected by symmetry.

For all of the crystals considered, the electron–ion term from the constant electron background alone is sufficient to counterbalance the corresponding ion–ion term. The oscillating electron background provides additional stability for these diagonal terms. We note that, for the complete stability hierarchy in the density nearly-free electron system, the dynamical matrices need to be studied, as well as additional effects, such as the electron per atom concentration and the band lowering at the Brillouin zone boundaries [50,51].

In the empty lattice approximation ($\tilde{u}\approx 0$), we find that cubic systems have positive diagonal harmonic terms of larger magnitude than hexagonal systems, and in particular the fcc structure has the largest diagonal harmonic term. This potentially concurs with the nearly-free limit *c* ≫ *a*_e_ and *c* ≫ *a* of the density tight-binding model. Furthermore, this matches observations in the periodic table, not only for the quintessential empty lattice case study, aluminium [52], but also nickel, copper, silver and gold [53]—all of which have an fcc structure.

In this section, we have shown that, considering diagonal harmonic terms with respect to the motion of a single ion, all ionic crystals are counterbalanced with the addition of a constant neutralizing background, and that a first-order oscillatory component to the background does not have a destabilizing effect. The fcc lattice has the largest such term, in agreement with many itinerant elemental solids.

## 6. Conclusion

In this paper, we have studied lattice instability of unconfined crystal structures at zero temperature in the bare ionic, density tight-binding and density nearly-free electron models. We analysed the diagonal matrices of force constants to expose instability and focused on the {cub, bcc, fcc, dia, hcp, dhcp} structures because of their prevalence in nature and distinctive properties.

In §3, we studied a bare one-component system of point Coulomb charges. First, we reviewed the history of the field, and noted that the bcc structure is special for being the stable crystal structure for both the low-density Wigner crystal and the high-density Coulomb crystal in the one-component plasma model. We then demonstrated that, in this regime, centrosymmetric cubic crystal structures can have no diagonal contribution to the dynamical matrix at quadratic order but instead an instability at fourth order, whereas all other crystal structures have an instability at second order. This is counter to the paradigmatic quasi-harmonic model of ions connected by springs [13–15]. These findings motivated us to continue and examine the preferred structure as we permit background charges to stabilize the system.

In §4, we stabilized the lattice through the addition of electron orbitals. We constructed a density tight-binding model, and found that in the extreme density tight-binding limit the bcc structure is the most stable, as suspected from the results and discussion in §3. We also showed that, if we tune the parameters to increase screening in our pseudopotential model of the nucleus and approach the nearly-free electron limit, the fcc structure is the stable ground state. This agrees with theoretical studies of unconfined three-dimensional Yukawa crystals in the literature. Finally, we report the second dominant phase to be hcp as we tune away from density tight binding, which accords to trends in the periodic table. The use of the density tight-binding model with the systematic analysis of terms has allowed us to combine the emergence of these three separate phases into a single framework.

In §5, we briefly examined the instability of crystal structures in the opposite limit, density nearly-free electrons, which is representative of many common metals. In this model, we found that the instability of every crystal structure is counterbalanced with the addition of a constant neutralizing background, and that a first-order oscillatory perturbation to the background does not have a destabilizing effect. We note here that a formal stability analysis would require the complete dynamical matrix. The most stable crystal structure in the empty lattice approximation according to this metric is fcc, agreeing not only with a limit of the density tight-binding model but also with several structures observed in the periodic table.

By investigating three simple cases, motivated by the absence of diagonal force constants in cubic Coulomb crystals, this paper highlights the connections and limitations of paradigmatic crystal models for stability. For many real materials, there are a plethora of important effects that need to be taken into consideration to determine the optimal lattice structure, e.g. temperature effects, the shape of the atomic orbitals, the precise band structure or the van der Waals interaction, which leaves scope for future work. However, we have identified minimal models to illustrate the interesting physical effects at play, shown how the density tight-binding and density nearly-free electron theories may be linked and connected historical theories of crystal structures at low energies. We hope that this closer look at the energies and force constants for these three models will instil a greater appreciation and understanding of the requirements for crystal stability, as well as its connection with lattice geometry.

## Supplementary Material

Supplementary Material

## Supplementary Material

Mathematica Scripts

## Footnotes

1 We use the terms ‘density tight-binding model’ and ‘density nearly-free electron model’ to emphasize the fact that this is in reference to the electron densities.

2 *J* = 0 without loss of generality.

3 This quantity would not be sufficient to quantify whether a system is stable.

4 Higher order (in)stabilities are, in all cases, weaker than those at lower orders.

5 Additionally, the successful quest to determine the intermediate physics arguably inspired the first application of importance-sampled diffusion Monte Carlo [34].

6 Odd power terms in the potential trivially vanish owing to symmetry.

7 See electronic supplementary material, section SIV.

## Data accessibility

The Mathematica scripts used to generate the data are provided as electronic supplementary material.
