# Supplementary material for: Absence of diagonal force constants in cubic Coulomb crystals
Source: Proc Math Phys Eng Sci. 2020 Dec 23;476(2244):20200518. doi: 10.1098/rspa.2020.0518 (PMC7776971; doi:10.1098/rspa.2020.0518)
Supplement: Supplementary Material [file rspa20200518supp1.pdf]

Supplementary Material: Absence of diagonal force constants in cubic Coulomb crystals

CONTENTS

SI. Proof that $\sum_\alpha \Phi_{I\alpha,0i\alpha} = 0$ for $I \neq 0$ 1

SII. Higher-order Derivative Test 2

SIII. Higher-order Matrices of Force Constants 2

SIV. Crystal Structures in the Periodic Table 3

SV. Numerical Model 3
- A. Ewald summation 3
- B. Cubic systems (cub, bcc, fcc, dia) 4
- C. Hexagonal systems (hcp, dhcp) 4

SVI. Details of the Density Tight-binding Model 6
- A. Definitions 7
  - 1. Wavefunction 7
  - 2. Electron cloud potential and density 9
  - 3. Ion potential and density 9
- B. Ion-ion contribution 9
- C. Electron-ion contribution 10
- D. Electron-electron contribution 10
- E. Pauli repulsion 11
- F. Crystal relaxation 11

SVII. Oscillatory Electron Density in the Density Nearly-free Electron Model 12

References 12

SI. PROOF THAT $\sum_\alpha \Phi_{I\alpha,0i\alpha} = 0$ FOR $I \neq 0$

The diagonal matrices of force constants, $\mathbf{\Phi}_I \equiv \Phi_{I\alpha,0i\beta}$, corresponding to the diagonal dynamical matrix, $D_{i\alpha,i\beta}$, are computed by Taylor expanding a crystal with respect to atom perturbations, as discussed in Sec. I. In order to demonstrate Poisson's law, $\sum_\alpha \Phi_{0i\alpha,0i\alpha} = 0$, it is sufficient to perturb only the central atom, since $\sum_\alpha \Phi_{0i\alpha,0i\alpha}$ corresponds to the Laplacian. In order to show $\sum_\alpha \Phi_{I i\alpha,0i\alpha} = 0$ for $I \neq 0$, however, we need to subsequently perturb a second atom to compute the mixed second derivatives $\Phi_{Ii\alpha,0i\alpha}$.

For symmetry, let us consider a crystal of atoms consisting of superimposed positive and negative charges, and choose to perturb the positive charges. After displacing the central positive charge by $\mathbf{u}_1 = (x_1, y_1, z_1)$, we subsequently displace a positive charge at $\mathbf{R}_I = (X, Y, Z)$ by $\mathbf{u}_2 = (x_2, y_2, z_2)$. The negative shadow charges remain fixed. In this notation, $\mathbf{R}_I$ denotes the separation vector between unit cells 0 and I in the crystal. Examining the symmetric interaction between these two atoms (four charges), yields the potential energy contribution

$$E_I = \frac{1}{|\mathbf{R}_I + (\mathbf{u}_2 - \mathbf{u}_1)|} - \frac{1}{|\mathbf{R}_I + \mathbf{u}_2|} - \frac{1}{|\mathbf{R}_I - \mathbf{u}_1|} + \frac{1}{|\mathbf{R}_I|} = \frac{1}{2}\mathbf{u}_1^\intercal \mathbf{\Phi}_I \mathbf{u}_2 + O(\text{cubic}),$$

where

$$\mathbf{\Phi}_I = \frac{2}{(X^2+Y^2+Z^2)^{5/2}} \begin{pmatrix} -2X^2+Y^2+Z^2 & -3XY & -3XZ \\ -3XY & X^2-2Y^2+Z^2 & -3YZ \\ -3XZ & -3YZ & X^2+Y^2-2Z^2 \end{pmatrix}.$$

The trace of this matrix is zero for any separation vector $\mathbf{R}_I$. Hence, the formula $\sum_\alpha \Phi_{Ii\alpha,0i\alpha} = 0$ holds for all integer $I \neq 0$ and any crystal structure.

SII. HIGHER-ORDER DERIVATIVE TEST

For a single-variable, real-valued and sufficiently differentiable function, f, let the first $(n-1)$ derivatives vanish such that

$$f'(c) = \cdots = f^{(n-1)}(c) = 0 \quad \text{and} \quad f^{(n)}(c) \neq 0,$$

where c is a constant in the domain of the function, and $n \in \mathbb{Z}^+$. In this case, the nth derivative may be used as a discriminant to determine the nature of the turning points.

If n is even:

- $f^{(n)}(c) < 0 \Rightarrow c$ is a local maximum,
- $f^{(n)}(c) > 0 \Rightarrow c$ is a local minimum.

If n is odd:

- $f^{(n)}(c) < 0 \Rightarrow c$ is a strictly decreasing point of inflection,
- $f^{(n)}(c) > 0 \Rightarrow c$ is a strictly increasing point of inflection.

Hence, this test can classify the critical points of f in all cases, provided $f^{(n)}(c) \neq 0$ for some value of n[S1].

The higher-order derivative test may be generalized to multi-dimensional problems. Denoting $D^{(p)}f$ as the pth-order multivariate derivative of f, it can be shown that under corresponding conditions:

- $D^{(p)}f(c)$ is negative definite $\Rightarrow c$ is a strict local maximum.
- $D^{(p)}f(c)$ is positive definite $\Rightarrow c$ is a strict local minimum,
- $D^{(p)}f(c)$ is indefinite $\Rightarrow c$ is a saddle point,
- $D^{(p)}f(c)$ is zero or semidefinite $\Rightarrow$ the test is inconclusive.

Note that, unlike the single-variable test, this test is not conclusive in all cases[S2].

SIII. HIGHER-ORDER MATRICES OF FORCE CONSTANTS

In this paper we consider the effect of displacing atoms originally at $\{\mathbf{R}^0_{Ii}\}$, on their nearest neighbors, with a energy E and general displacements $\{\mathbf{R}_{Ii}\}$. We may expand the energy such that:

$$\begin{aligned} E(\{\mathbf{R}_{Ii}\}) =& E(\{\mathbf{R}^0_{Ii}\}) + \sum_{I\alpha} \left.\frac{\partial E}{\partial u_{I\alpha}}\right|_{\mathbf{u}=\mathbf{0}} u_{I\alpha} + \frac{1}{2!}\sum_{I\alpha}\sum_{J\beta} \Phi_{I\alpha,J\beta} u_{I\alpha} u_{J\beta} \\ &+ \frac{1}{3!}\sum_{I\alpha}\sum_{J\beta}\sum_{K\gamma} \left.\frac{\partial^3 E}{\partial u_{I\alpha}\partial u_{J\beta}\partial u_{K\gamma}}\right|_{\mathbf{u}=\mathbf{0}} u_{I\alpha} u_{J\beta} u_{K\gamma} \\ &+ \frac{1}{4!}\sum_{I\alpha}\sum_{J\beta}\sum_{K\gamma}\sum_{Ll\delta} X_{I\alpha,J\beta,K\gamma,Ll\delta} u_{I\alpha} u_{J\beta} u_{K\gamma} u_{Ll\delta} + \ldots, \end{aligned}$$

where $\{I, J, K, L\}$ are unit cell indices, $\{i, j, k, l\}$ are basis atom indices, and $\{\alpha, \beta, \gamma, \delta\}$ are Cartesian directions. As stated in the main text, translational invariance allows us to consider $\Phi_{Ii\alpha,0j\beta}$ and $X_{Ii\alpha,0j\beta,Kk\gamma,Ll\delta}$ without loss of generality. Furthermore, exploiting the symmetry of the system, we additionally contract the fourth-order matrix of force constants such that $X_{Ii\alpha,0j\beta,Kk\gamma,Ll\delta}\delta^0_K\delta^K_L\delta^\alpha_\beta\delta^\gamma_\delta = X_{Ii\alpha,0j\alpha,0k\gamma,0l\gamma}$, which allows us to write the diagonal force constant matrices analogously as $\mathbf{\Phi}_I \equiv \Phi_{Ii\alpha,0i\beta}$ and $\mathbf{X}_I \equiv X_{Ii\alpha,0i\beta}$. Both of these matrices are symmetric in (α, β).

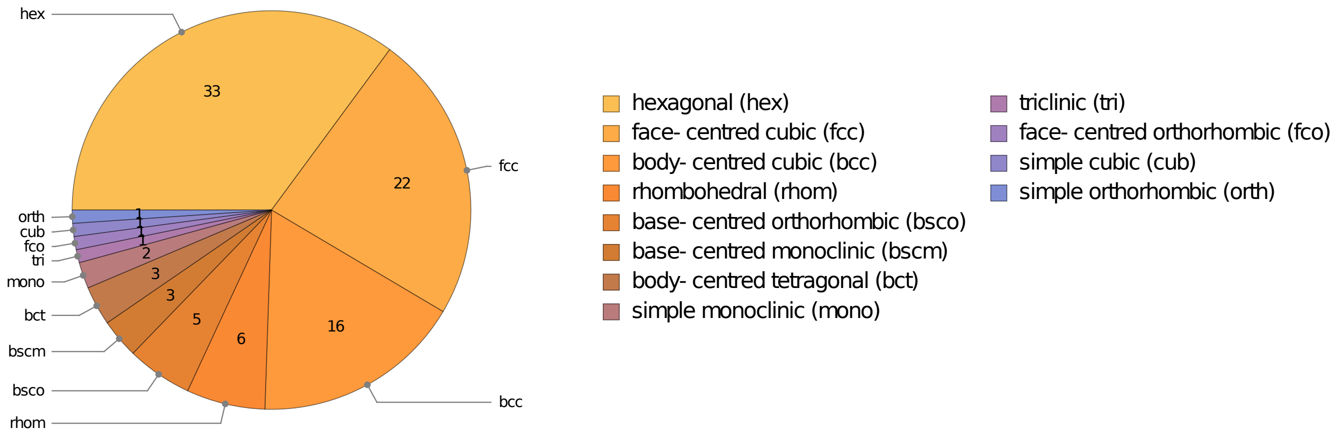

FIG. S1. Breakdown of crystal structures (by Bravais lattice) for the most thermodynamically stable allotropes of elements in the periodic table, at standard temperature and pressure. The number of elements exhibiting each crystal structure is given in the corresponding section of the chart. Crystal structure data is provided for an unambiguous subset containing 94 out of the 118 elements, using Mathematica's ElementData function[S3].

SIV. CRYSTAL STRUCTURES IN THE PERIODIC TABLE

Sufficiently stable elements in the periodic table may be grouped in accordance with their crystal structure. A breakdown of the crystal structures (by Bravais lattice) in the periodic table is presented in Fig. S1. In the cases where an element exhibits multiple crystal structures at standard temperature and pressure, the most thermodynamically stable allotrope is given.

In three-dimensions, all crystal structures are derived from fourteen possible Bravais lattices. However, some of the derived crystal structures are worth studying separately, either due to their ubiquity (e.g. in the case of the hcp structure: the most common crystal structure in nature) or interesting properties (e.g. in the case of diamond). The cub, bcc, fcc, dia, hcp, and dhcp crystal structures are studied in particular in this paper because they only have one free parameter: the lattice constant. Furthermore, this group of crystal structures accounts for approximately three quarters of the known crystal structures in the periodic table.

SV. NUMERICAL MODEL

In this section, we outline the numerical details of how the crystal structure summations were performed. In the interests of clarity, we use simplified notation in this section and consider a one-component crystal with the displacement of a single ion at the origin by a displacement vector $\mathbf{R}$ and total potential energy E.

A. Ewald summation

Ewald summation is the standard method to compute Coulomb interactions in infinite periodic systems, such as crystals[S4]. The method works by splitting the Coulomb potential into a singular short-range term, which is evaluated in real space, and a continuous long-range term, which is evaluated in momentum space. The split is performed such that $V(r) = 1/r = f(\alpha r)/r - (1 - f(\alpha r))/r$, where $f(r) = \mathrm{erf}(r)$ is the error function, $1 - f(r) = \mathrm{erfc}(r)$ is the complementary error function, and $\alpha > 0$ is the Ewald splitting parameter. The error function is typically chosen because it corresponds to a Gaussian spreading function for the point charges and its Fourier coefficients are known analytically.

In the 3D periodic Coulomb problem, the total potential may be written as

$$E = \sum_{I} \sum_{i<j} \frac{1}{|\mathbf{R}_I + \mathbf{r}_{ij}|},$$

where $\mathbf{r}_{ij} \equiv \mathbf{r}_i - \mathbf{r}_j$ and we sum over all distinct pairs of identical unit charges. Rewriting the total potential in the Ewald formalism yields

$$E = \frac{1}{2}\sum_I{}' \sum_{i,j} \frac{\mathrm{erfc}(\alpha|\mathbf{R}_I + \mathbf{r}_{ij}|)}{|\mathbf{R}_I + \mathbf{r}_{ij}|} + \frac{1}{2}\sum_{I\neq 0}\sum_{i,j} \frac{4\pi e^{-k_I^2/(4\alpha^2)}}{k_I^2 a^3} e^{-\mathrm{i}\mathbf{k}\cdot\mathbf{r}_{ij}} - \frac{\alpha N_\mathrm{u}}{\sqrt{\pi}},$$

where $\mathbf{k}_I = 2\pi\mathbf{R}_I/a$ is the momentum, a is the unit cell length, and N_u is the number of ions per unit cell[S5]. The first term is short-range and evaluated directly by summing concentric shells, as discussed in the following sections. The second term is summed analogously in reciprocal space with a cutoff empirically set such that the Ewald splitting parameter $\alpha \approx 2$[S6]. The final term is the self energy, which cancels the corresponding contribution from the momentum sum. Depending on the exact parameters chosen for the cutoffs, the scaling of the classical Ewald summation is between $O(N^{3/2})$ and $O(N^2)$, where N is the number of ions in the system.

To demonstrate the convergence of the classical Ewald method, we show in Fig. S2 the computation of the trace of the diagonal matrices of force constants with respect to the motion of a single ion, $\Phi_{I\alpha,I\beta}$, against the number of shells in the real-space summation, n, for a variety of perturbation centers, $\mathbf{R}_I = i_\mathrm{x}\mathbf{a} + i_\mathrm{y}\mathbf{b} + i_\mathrm{z}\mathbf{c}$, where $\{\mathbf{a}, \mathbf{b}, \mathbf{c}\}$ are the crystal basis vectors. For cubic crystals, it can be seen that $\sum_\alpha \Phi_{I\alpha,I\alpha} = 0$ by Poisson's law and $\Phi_{I\alpha,I\alpha} = 0$ by symmetry. For hexagonal crystals, we again note that $\sum_\alpha \Phi_{I\alpha,I\alpha} = 0$ by Poisson's law, however the diagonal elements $\Phi_{I\alpha,I\alpha} \neq 0$. In this paper, we sum shells up to and including $n = 8$, which is deep into the converged region.

B. Cubic systems (cub, bcc, fcc, dia)

In this paper, we consider unit cells with an ion situated at the origin in all cases. We refer to these as *origin-centric* unit cells, and we choose these in order to minimize finite system size error when summing radially outwards over many shells, as well as to simplify the computations. The unit cell for the simple cubic crystal consists of one ion situated at the origin. The unit cells for the bcc, fcc, and dia crystal lattices are shown in Fig. S3.

In order to sum to n shells, we include all ions in units cells whose origins are situated within a radius of n lattice constants, as illustrated in Fig. S4. We continue to sum in this fashion until the properties of interest, such as the diagonal force constant matrices, converge to the desired precision.

The coordinates of the unit cell sites for these cubic systems is shown in Table S1a and the corresponding potentials are given in Table S1b. Hence, the summation over n shells may be written explicitly as

$$E_C = \sum_I V_C(\mathbf{R}_I - \mathbf{R}) = \sum_{i_\mathrm{x}^2+i_\mathrm{y}^2+i_\mathrm{z}^2 \le n^2} V_C\left(a\begin{pmatrix} i_\mathrm{x} \\ i_\mathrm{y} \\ i_\mathrm{z}\end{pmatrix} - \begin{pmatrix} X \\ Y \\ Z\end{pmatrix}\right) - V\left(\begin{pmatrix} X \\ Y \\ Z\end{pmatrix}\right),$$

where $C \in \{\text{cub, bcc, fcc, dia}\}$ denotes the cubic crystal structure under consideration, and $\{i_\mathrm{x}, i_\mathrm{y}, i_\mathrm{z}\}$ are integers. The complete summation, including the long-range contribution, yields the potential energy of displacing the ion at the origin to a position $\mathbf{R}$. The converged expression can then be expanded to quadratic order in $\mathbf{R}$, for example, to extract the diagonal force constant matrix.

C. Hexagonal systems (hcp, dhcp)

Hexagonal systems are treated in an analogous fashion to cubic systems, except now more care is needed since the vectors to neighboring unit cells are not orthogonal. The origin-centric unit cells for the hcp and dhcp crystal lattices are shown in Figs. S5a & S5b and the corresponding displacement vectors and potentials are presented in Table S2. Hence for these systems, the (unnormalized) basis set, to go from one unit cell to another, may be denoted as

$$\{\mathbf{a}, \mathbf{b}, \mathbf{c}\} = \frac{a}{2}\left\{\begin{pmatrix} 3 \\ -\sqrt{3} \\ 0\end{pmatrix}, \begin{pmatrix} 3 \\ \sqrt{3} \\ 0\end{pmatrix}, \begin{pmatrix} 0 \\ 0 \\ \frac{4\sqrt{6}}{3}\end{pmatrix}\right\}, \tag{S1}$$

where a is the lattice constant in the xy-plane. In this case, the summation over n shells may be explicitly written as

$$E_H = \sum_I V_H(\mathbf{R}_I - \mathbf{R}) = \sum_{\left(\frac{\sqrt{3}}{2}(i_\mathrm{x}+i_\mathrm{y})\right)^2 + \left(\frac{i_\mathrm{y}-i_\mathrm{x}}{2}\right)^2 + i_\mathrm{z}^2 \le n^2} V_H\left(\frac{a}{2}\begin{pmatrix} 3(i_\mathrm{x}+i_\mathrm{y}) \\ \sqrt{3}(i_\mathrm{y}-i_\mathrm{x}) \\ \frac{4\sqrt{6}}{3}i_\mathrm{z}\end{pmatrix} - \begin{pmatrix} X \\ Y \\ Z\end{pmatrix}\right) - V\left(\begin{pmatrix} X \\ Y \\ Z\end{pmatrix}\right),$$

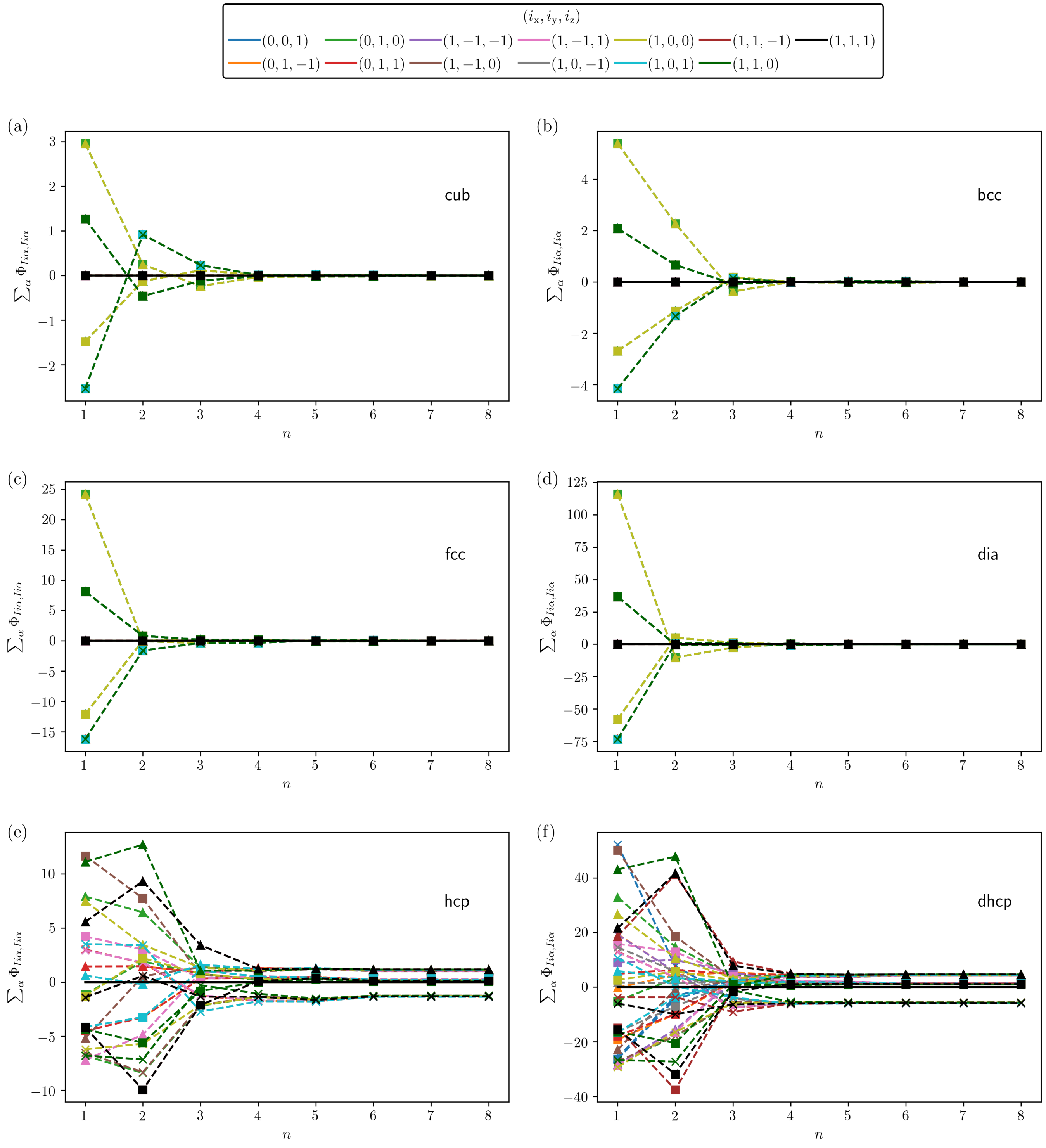

FIG. S2. The trace of the diagonal matrix of force constants, $\Phi_{Ii\alpha,Ii\beta}$, for all $\mathbf{R}_I$ satisfying $0 < |\mathbf{R}_I| \leq \sqrt{3}$ – excluding those related by inversion symmetry $\mathbf{R}_I \leftrightarrow -\mathbf{R}_I$ – against the number of shells in the summation, n, for the (a) cub, (b) bcc, (c) fcc, (d) dia, (e) hcp, and (f) dhcp crystal structures. To explicitly show the convergence, we also present the diagonal matrix elements (dashed lines): $\Phi_{Ii0,Ii0}$ (triangles), $\Phi_{Ii1,Ii1}$ (squares), and $\Phi_{Ii2,Ii2}$ (crosses) for each $\mathbf{R}_I$ and crystal structure.

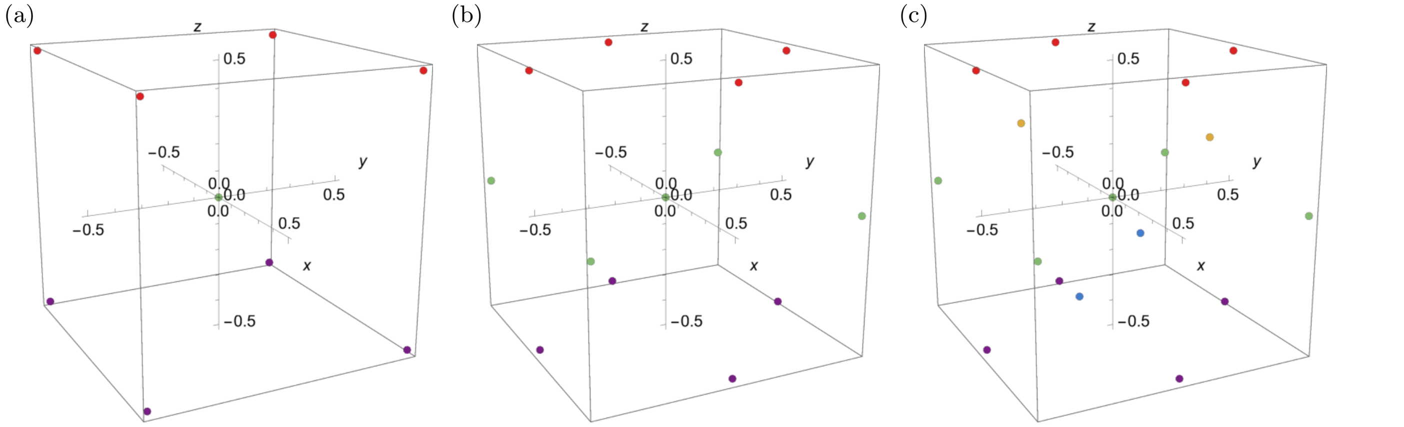

FIG. S3. Origin-centric unit cells for the (a) bcc, (b) fcc, and (c) dia, crystal structures. These structures have two, four, and eight ions per unit cell, respectively. All lengths are given in units of the lattice constant, and the coloring distinguishes the position along the z-axis. The displacement vectors for these plots are given in Table S1a.

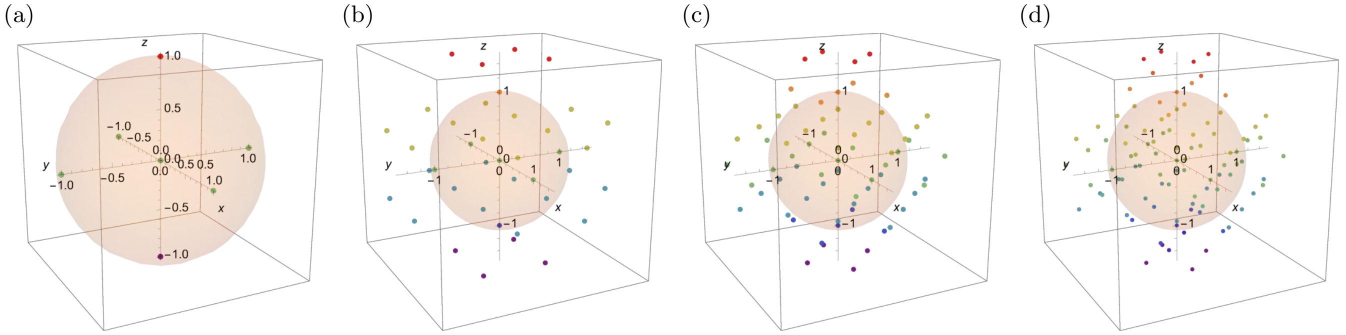

FIG. S4. Illustration of the points included in a one-shell summation of the (a) cub, (b) bcc, (c) fcc, and (d) dia, crystal structures. All points from the nearest-neighbor unit cells are plotted. The centers of neighboring unit cells lie within a unit sphere (light orange). All lengths are given in units of the lattice constant, and the coloring of points distinguishes their position along the z-axis.

where $H \in \{\text{hcp}, \text{dhcp}\}$ denotes the hexagonal crystal structure under consideration, and $\{i_\text{x}, i_\text{y}, i_\text{z}\}$ are integers. Figures S5c & S5d show the sites included in these summations up to eight shells, which is typically the number at which the desired precision converged. Note the approximate spherical symmetry of these systems.

SVI. DETAILS OF THE DENSITY TIGHT-BINDING MODEL

In this section, we outline the details of the density tight-binding configuration. As in Sec. SV, we use simplified notation for clarity. In our model, we have a crystal of ions with tightly-bound electrons at each site. We consider each atom to be composed of a pseudopotential, which takes into account the potential of the nucleus screened by the inner electrons, and one outermost electron. As before, we employ the classical Ewald method to incorporate the long-range contribution.

(a) Displacement Vectors

Crystal	Plane	Displacement Vectors of Sites in the Unit Cell
bcc	$z = a/2$	$\mathbf{r}_1^{\rm bcc} = \frac{a}{2}(1,1,1)$, $\mathbf{r}_2^{\rm bcc} = \frac{a}{2}(-1,1,1)$, $\mathbf{r}_3^{\rm bcc} = \frac{a}{2}(1,-1,1)$, $\mathbf{r}_4^{\rm bcc} = \frac{a}{2}(-1,-1,1)$,
	$z = -a/2$	$\mathbf{r}_5^{\rm bcc} = \frac{a}{2}(1,1,-1)$, $\mathbf{r}_6^{\rm bcc} = \frac{a}{2}(1,-1,-1)$, $\mathbf{r}_7^{\rm bcc} = \frac{a}{2}(-1,1,-1)$, $\mathbf{r}_8^{\rm bcc} = \frac{a}{2}(-1,-1,-1)$
fcc	$z = 0$	$\mathbf{r}_1^{\rm fcc} = \frac{a}{2}(1,1,0)$, $\mathbf{r}_2^{\rm fcc} = \frac{a}{2}(-1,1,0)$, $\mathbf{r}_3^{\rm fcc} = \frac{a}{2}(1,-1,0)$, $\mathbf{r}_4^{\rm fcc} = \frac{a}{2}(-1,-1,0)$,
	$y = 0$	$\mathbf{r}_5^{\rm fcc} = \frac{a}{2}(1,0,1)$, $\mathbf{r}_6^{\rm fcc} = \frac{a}{2}(-1,0,1)$, $\mathbf{r}_7^{\rm fcc} = \frac{a}{2}(1,0,-1)$, $\mathbf{r}_8^{\rm fcc} = \frac{a}{2}(-1,0,-1)$,
	$x = 0$	$\mathbf{r}_9^{\rm fcc} = \frac{a}{2}(0,1,1)$, $\mathbf{r}_{10}^{\rm fcc} = \frac{a}{2}(0,-1,1)$, $\mathbf{r}_{11}^{\rm fcc} = \frac{a}{2}(0,1,-1)$, $\mathbf{r}_{12}^{\rm fcc} = \frac{a}{2}(0,-1,-1)$
dia	$z = 0$	$\mathbf{r}_1^{\rm dia} = \frac{a}{8}(4,4,0)$, $\mathbf{r}_2^{\rm dia} = \frac{a}{8}(-4,4,0)$, $\mathbf{r}_3^{\rm dia} = \frac{a}{8}(4,-4,0)$, $\mathbf{r}_4^{\rm dia} = \frac{a}{8}(-4,-4,0)$,
	$y = 0$	$\mathbf{r}_5^{\rm dia} = \frac{a}{8}(4,0,4)$, $\mathbf{r}_6^{\rm dia} = \frac{a}{8}(-4,0,4)$, $\mathbf{r}_7^{\rm dia} = \frac{a}{8}(4,0,-4)$, $\mathbf{r}_8^{\rm dia} = \frac{a}{8}(-4,0,-4)$,
	$x = 0$	$\mathbf{r}_9^{\rm dia} = \frac{a}{8}(0,4,4)$, $\mathbf{r}_{10}^{\rm dia} = \frac{a}{8}(0,-4,4)$, $\mathbf{r}_{11}^{\rm dia} = \frac{a}{8}(0,4,-4)$, $\mathbf{r}_{12}^{\rm dia} = \frac{a}{8}(0,-4,-4)$,
	$z = a/4$	$\mathbf{r}_{13}^{\rm dia} = \frac{a}{8}(2,2,2)$, $\mathbf{r}_{14}^{\rm dia} = \frac{a}{8}(-2,-2,2)$,
	$y = a/4$	$\mathbf{r}_{15}^{\rm dia} = \frac{a}{8}(-2,2,-2)$,
	$x = a/4$	$\mathbf{r}_{16}^{\rm dia} = \frac{a}{8}(2,-2,-2)$

(b) Potentials

Crystal	Atoms per Unit Cell	Potential
bcc	2	$V_{\rm bcc}(\mathbf{R}) = V(\mathbf{R}) + \frac{1}{8}\sum_{i=1}^{8} V(\mathbf{R}+\mathbf{r}_i^{\rm bcc})$
fcc	4	$V_{\rm fcc}(\mathbf{R}) = V(\mathbf{R}) + \frac{1}{4}\sum_{i=1}^{12} V(\mathbf{R}+\mathbf{r}_i^{\rm fcc})$
dia	8	$V_{\rm dia}(\mathbf{R}) = V(\mathbf{R}) + \frac{1}{4}\sum_{i=1}^{12} V(\mathbf{R}+\mathbf{r}_i^{\rm dia}) + \sum_{i=13}^{16} V(\mathbf{R}+\mathbf{r}_i^{\rm dia})$

TABLE S1. (a) Displacement vectors for sites in a unit cell, and (b) corresponding unit cell potentials, for the bcc, fcc, and dia crystal structures. For the displacement vectors, the site at the origin is omitted and all vectors are given in terms of the lattice constant, a.

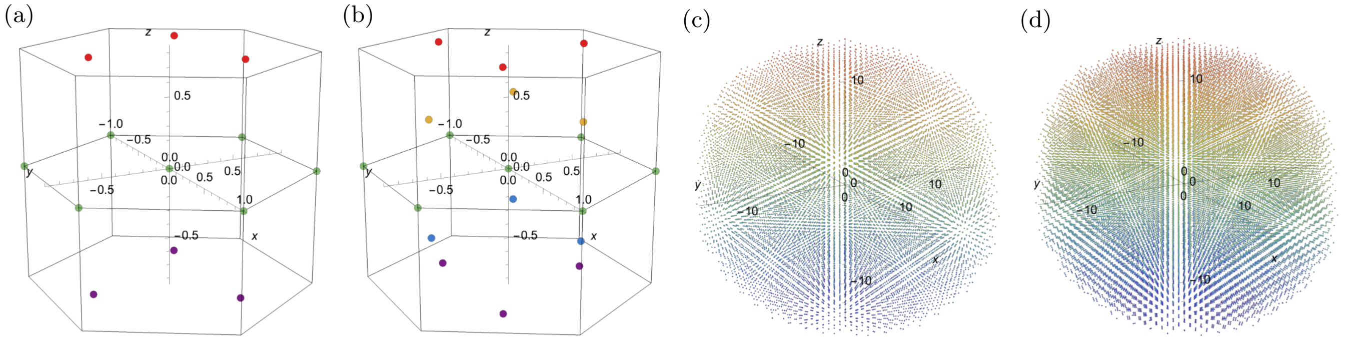

FIG. S5. [(a), (b)] Origin-centric unit cells for the (a) hcp, and (b) dhcp, crystal structures. These structures have six and twelve ions per unit cell, respectively. [(c), (d)] Illustrations of the (c) hcp, and (d) dhcp, crystal structures plotted up to eight shells. All lengths are given in units of the lattice constant, and the coloring distinguishes the position along the z-axis. The displacement vectors for these plots are given in Table S2.

A. Definitions

1. *Wavefunction*

We start by taking a simplified ansatz for the wavefunction of the valence electron orbital under the potential of the ion:

$$\Psi(\mathbf{R}; c, a_{\rm e}) = A\sqrt{\frac{1}{1+\exp\left(\frac{2(|\mathbf{R}|-c)}{a_{\rm e}}\right)}},$$

where A is a normalization constant, $a_{\rm e} \ll R_I$ is the width of the valence electron cloud, and $0 \le c < a_{\rm e}$ is the width of the core electron cloud. We choose this ansatz so that the electron density is analytically well behaved in

(a) Displacement Vectors

Crystal	Plane	Displacement Vectors of Sites in the Unit Cell
hcp	$z=0$	$\mathbf{r}_1^{\text{hcp}}=a(1,0,0)$, $\mathbf{r}_2^{\text{hcp}}=\frac{a}{2}(1,\sqrt{3},0)$, $\mathbf{r}_3^{\text{hcp}}=\frac{a}{2}(-1,\sqrt{3},0)$,
		$\mathbf{r}_4^{\text{hcp}}=a(-1,0,0)$, $\mathbf{r}_5^{\text{hcp}}=\frac{a}{2}(-1,-\sqrt{3},0)$, $\mathbf{r}_6^{\text{hcp}}=\frac{a}{2}(1,-\sqrt{3},0)$,
	$z=2\sqrt{6}/3$	$\mathbf{r}_7^{\text{hcp}}=\frac{a}{6}(3,\sqrt{3},2\sqrt{6})$, $\mathbf{r}_8^{\text{hcp}}=\frac{a}{6}(-3,\sqrt{3},2\sqrt{6})$, $\mathbf{r}_9^{\text{hcp}}=\frac{a}{3}(0,-\sqrt{3},\sqrt{6})$,
	$z=-2\sqrt{6}/3$	$\mathbf{r}_{10}^{\text{hcp}}=\frac{a}{6}(3,\sqrt{3},-2\sqrt{6})$, $\mathbf{r}_{11}^{\text{hcp}}=\frac{a}{6}(-3,\sqrt{3},-2\sqrt{6})$, $\mathbf{r}_{12}^{\text{hcp}}=\frac{a}{3}(0,-\sqrt{3},-\sqrt{6})$
dhcp	$z=0$	$\mathbf{r}_1^{\text{dhcp}}=a(1,0,0)$, $\mathbf{r}_2^{\text{dhcp}}=\frac{a}{2}(1,\sqrt{3},0)$, $\mathbf{r}_3^{\text{dhcp}}=\frac{a}{2}(-1,\sqrt{3},0)$,
		$\mathbf{r}_4^{\text{dhcp}}=a(-1,0,0)$, $\mathbf{r}_5^{\text{dhcp}}=\frac{a}{2}(-1,-\sqrt{3},0)$, $\mathbf{r}_6^{\text{dhcp}}=\frac{a}{2}(1,-\sqrt{3},0)$,
	$z=\sqrt{6}/6$	$\mathbf{r}_7^{\text{dhcp}}=\frac{a}{6}(3,\sqrt{3},\sqrt{6})$, $\mathbf{r}_8^{\text{dhcp}}=\frac{a}{6}(-3,\sqrt{3},\sqrt{6})$, $\mathbf{r}_9^{\text{dhcp}}=\frac{a}{3}(0,-\sqrt{3},\frac{\sqrt{6}}{2})$,
	$z=-\sqrt{6}/6$	$\mathbf{r}_{10}^{\text{dhcp}}=\frac{a}{6}(3,\sqrt{3},-\sqrt{6})$, $\mathbf{r}_{11}^{\text{dhcp}}=\frac{a}{6}(-3,\sqrt{3},-\sqrt{6})$, $\mathbf{r}_{12}^{\text{dhcp}}=\frac{a}{3}(0,-\sqrt{3},-\frac{\sqrt{6}}{2})$,
	$z=\sqrt{6}/3$	$\mathbf{r}_{13}^{\text{dhcp}}=\frac{a}{6}(3,-\sqrt{3},2\sqrt{6})$, $\mathbf{r}_{14}^{\text{dhcp}}=\frac{a}{6}(-3,-\sqrt{3},2\sqrt{6})$, $\mathbf{r}_{15}^{\text{dhcp}}=\frac{a}{3}(0,\sqrt{3},\sqrt{6})$,
	$z=-\sqrt{6}/3$	$\mathbf{r}_{16}^{\text{dhcp}}=\frac{a}{6}(3,-\sqrt{3},-2\sqrt{6})$, $\mathbf{r}_{17}^{\text{dhcp}}=\frac{a}{6}(-3,-\sqrt{3},-2\sqrt{6})$, $\mathbf{r}_{18}^{\text{dhcp}}=\frac{a}{3}(0,\sqrt{3},-\sqrt{6})$

(b) Potentials

Crystal	Atoms per Unit Cell	Potential
hcp	6	$V_{\text{hcp}}(\mathbf{R})=V(\mathbf{R})+\frac{1}{3}\sum_{i=1}^{6}V(\mathbf{R}+\mathbf{r}_i^{\text{hcp}})+\frac{1}{2}\sum_{i=7}^{12}V(\mathbf{R}+\mathbf{r}_i^{\text{hcp}})$
dhcp	12	$V_{\text{dhcp}}(\mathbf{R})=V(\mathbf{R})+\frac{1}{3}\sum_{i=1}^{6}V(\mathbf{R}+\mathbf{r}_i^{\text{dhcp}})+\sum_{i=7}^{12}V(\mathbf{R}+\mathbf{r}_i^{\text{dhcp}})+\frac{1}{2}\sum_{i=13}^{18}V(\mathbf{R}+\mathbf{r}_i^{\text{dhcp}})$

TABLE S2. (a) Displacement vectors for sites in a unit cell, and (b) corresponding unit cell potentials, for the hcp and dhcp crystal structures. For the displacement vectors, the site at the origin is omitted and all vectors are given in terms of the lattice constant, a.

(a) (b)

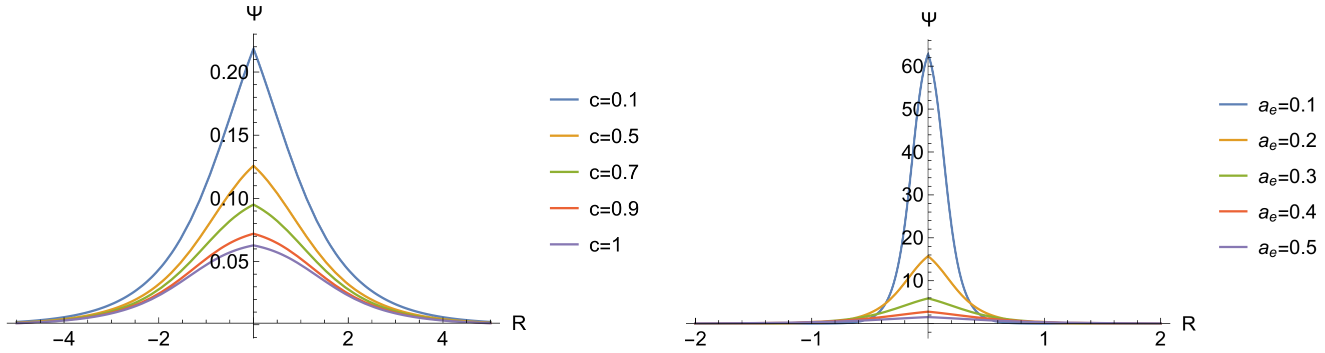

FIG. S6. Plots of the normalized wavefunction of the valence electron under the pseudopotential of the ion, Ψ. The behavior of the wavefunction is shown as we (a) vary c with $a_{\text{e}}=1$, and (b) vary a_{e} with $c=0.1$.

subsequent calculations, and that in the limit of vanishing radius and large distances we recover the wavefunction of a particle in a Dirac delta potential well:

$$\lim_{c\ll a_{\text{e}}\ll|\mathbf{R}|}\Psi\propto e^{-|\mathbf{R}|/a_{\text{e}}}. \tag{S2}$$

This is the limit around which we will expand in the following sections. Plots of this wavefunction are shown in Fig. S6. Since it is not possible to analytically derive an expression for the normalized wavefunction, we expand the probability density, $|\Psi|^2$ up to first order in the small parameter (c/a_{e}) and then solve the normalization condition $\int_{-\infty}^{\infty}|\Psi|^2\text{d}\mathbf{R}=a_0/a_{\text{e}}$, where a_0 is the Bohr radius. This yields a normalization constant

$$A(c,a_{\text{e}})=\frac{2\sqrt{a_0}\left(9a_{\text{e}}\zeta(3)-c\pi^2\right)}{9a_{\text{e}}^3\sqrt{3\pi}\zeta(3)^{3/2}}+O\left[\left(\frac{c}{a_{\text{e}}}\right)^2\right],$$

where $\zeta(3)$ is Apéry's constant.

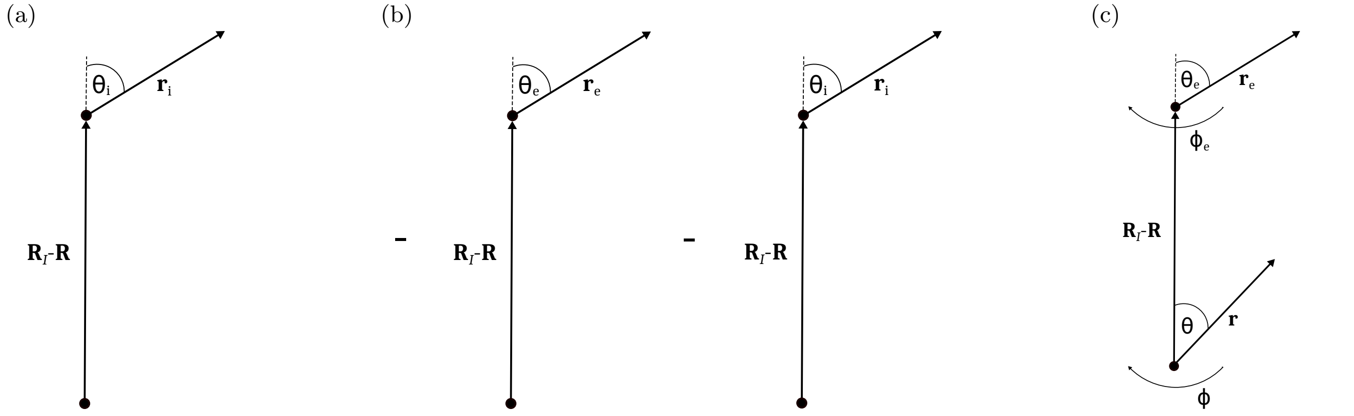

FIG. S7. Diagrams corresponding to the (a) ion-ion, (b) electron-ion, and (c) electron-electron contribution calculations. The displacement vector between ions, $\mathbf{R} - \mathbf{R}_I$, is oriented along the north pole, and the polar and azimuthal angles are defined in the range $0 \leq \theta < \pi$ and $0 \leq \phi < 2\pi$, respectively.

2. Electron cloud potential and density

The valence electron cloud (which we denote using a capital 'E') has a potential given by the Coulomb potential of the single electron, $V_\mathrm{e}(\mathbf{R}) = |\mathbf{R}|^{-1}$, integrated over the density distribution of the electron cloud:

$$V_\mathrm{E}(\mathbf{R}; c, a_\mathrm{e}) = \int V_\mathrm{e}(\mathbf{R} + \mathbf{r}_\mathrm{e})\rho_\mathrm{E}(\mathbf{r}_\mathrm{e}; c, a_\mathrm{e})\,\mathrm{d}\mathbf{r}_\mathrm{e},$$

where we calculate the density of the electron cloud using the normalized wavefunction defined in Sec. SVI A 1:

$$\rho_\mathrm{E}(\mathbf{r}_\mathrm{e}; c, a_\mathrm{e}) = |\Psi(\mathbf{r}_\mathrm{e}; c, a_\mathrm{e})|^2.$$

3. Ion potential and density

The ion potential is obtained by solving the time-independent Schrödinger equation and subtracting the energy constant, such that

$$V_\mathrm{i}(\mathbf{R}; c, a_\mathrm{e}) = -a_0\left(\frac{1}{\Psi}\frac{\nabla^2}{2}\Psi - \lim_{|\mathbf{R}|\to\infty}\left(\frac{1}{\Psi}\frac{\nabla^2}{2}\Psi\right)\right).$$

The ion density is then subsequently obtained from Poisson's equation:

$$\rho_\mathrm{i}(\mathbf{r}_\mathrm{i}; c, a_\mathrm{e}) = -\frac{\nabla^2 V_\mathrm{i}(\mathbf{r}_\mathrm{i}; c, a_\mathrm{e})}{4\pi}.$$

Note that due to the norm conserving property of our wavefunction ansatz, the ion density satisfies the normalization condition $\int_0^\infty \rho_\mathrm{i}(r_\mathrm{i}, c, a_\mathrm{e}) 4\pi r_\mathrm{i}^2 \mathrm{d}r_\mathrm{i} = a_0/a_\mathrm{e}$ up to first order in (c/a_e).

B. Ion-ion contribution

First, we calculate the repulsive potential felt by an ion at position $\mathbf{R}_I$ due to an ion being displaced from the origin to a position $\mathbf{R}$. An illustration of the set-up is shown in Fig. S7a. Note that we orient the displacement vector between the two ions along the north pole to simplify the calculations. In order to calculate the ion-ion potential for the whole system we then sum over all distinct atoms, such that

$$E^\mathrm{BO}_\text{i-i}(\mathbf{R}; c, a_\mathrm{e}) = \sum_I \int V_\mathrm{i}(\mathbf{R}_I - \mathbf{R} + \mathbf{r}_\mathrm{i}; c, a_\mathrm{e})\rho_\mathrm{i}(\mathbf{r}_\mathrm{i}; c, a_\mathrm{e})\,\mathrm{d}\mathbf{r}_\mathrm{i}.$$

Rewriting the ion potential in terms of the scalar variables defined in Fig. S7a, such that $V_\mathrm{i}(|\mathbf{R}_I - \mathbf{R}|, \{r_\mathrm{i}, \theta_\mathrm{i}\}; c, a_\mathrm{e})$, we may Taylor expand the ion potential up to leading order in $(r_\mathrm{i}/|\mathbf{R}_I - \mathbf{R}|)$:

$$E^{\mathrm{BO}}_{\text{i-i}}(\mathbf{R}; c, a_\mathrm{e}) = \sum_I V_\mathrm{i}(|\mathbf{R}_I - \mathbf{R}|; c, a_\mathrm{e}) \underbrace{\int \rho_\mathrm{i}(\mathbf{r}_\mathrm{i}; c, a_\mathrm{e})\, \mathrm{d}\mathbf{r}_\mathrm{i}}_{a_0/a} + 2\pi \sum_I \int_{r_\mathrm{i}=0}^{\infty} \int_{\theta_\mathrm{i}=0}^{\pi} \left(\frac{\partial^2 V_\mathrm{i}}{\partial r_\mathrm{i}^2}\right) r_\mathrm{i}^4 \rho_\mathrm{i}(r_\mathrm{i}; c, a_\mathrm{e}) \sin(\theta_\mathrm{i})\, \mathrm{d}\theta_\mathrm{i} \mathrm{d}r_\mathrm{i} + O\left[\left(\frac{r_\mathrm{i}}{|\mathbf{R}_I - \mathbf{R}|}\right)^3\right].$$

Note that the first-order term in the expansion vanishes by symmetry. Hence the final expression for the ion-ion contribution is derived accurate to first order in (c/a_e) and second order in $(r_\mathrm{i}/|\mathbf{R}_I - \mathbf{R}|)$. Taken together, this forms the leading-order analytical expansion about the density tight-binding limit introduced in Sec. SVI A 1.

C. Electron-ion contribution

The next contribution is that due to the electron-ion interaction. There are attractive potentials felt by the electron cloud due to the ions, as well as those felt by the ion due to the electron clouds. A sketch of this scenario is shown in Fig. S7b, where the minus signs indicate that this is an attractive interaction. As in the previous section, we set up the general form of the electron-ion contribution as

$$E^{\mathrm{BO}}_{\text{e-i}}(\mathbf{R}; c, a_\mathrm{e}) = -\sum_I \int V_\mathrm{i}(\mathbf{R}_I - \mathbf{R} + \mathbf{r}_\mathrm{e}; c, a_\mathrm{e}) \rho_\mathrm{E}(\mathbf{r}_\mathrm{e}; c, a_\mathrm{e})\, \mathrm{d}\mathbf{r}_\mathrm{e} - \sum_I \int V_\mathrm{E}(\mathbf{R}_I - \mathbf{R} + \mathbf{r}_\mathrm{i}; c, a_\mathrm{e}) \rho_\mathrm{i}(\mathbf{r}_\mathrm{i}; c, a_\mathrm{e})\, \mathrm{d}\mathbf{r}_\mathrm{i}.$$

It can be shown, either by symmetry or integration by parts, that this expression reduces to

$$E^{\mathrm{BO}}_{\text{e-i}}(\mathbf{R}; c, a_\mathrm{e}) = -2 \sum_I \int V_\mathrm{i}(\mathbf{R}_I - \mathbf{R} + \mathbf{r}_\mathrm{e}; c, a_\mathrm{e}) \rho_\mathrm{E}(\mathbf{r}_\mathrm{e}; c, a_\mathrm{e})\, \mathrm{d}\mathbf{r}_\mathrm{e}.$$

Rewriting the ion potential in terms of scalar variables, as before, we may Taylor expand up to leading order in $(r_\mathrm{e}/|\mathbf{R}_I - \mathbf{R}|)$:

$$E^{\mathrm{BO}}_{\text{e-i}}(\mathbf{R}; c, a_\mathrm{e}) = -2 \sum_I V_\mathrm{i}(|\mathbf{R}_I - \mathbf{R}|; c, a_\mathrm{e}) \underbrace{\int \rho_\mathrm{E}(\mathbf{r}_\mathrm{e}; c, a_\mathrm{e})\, \mathrm{d}\mathbf{r}_\mathrm{e}}_{a_0/a} - 4\pi \sum_I \int_{r_\mathrm{e}=0}^{\infty} \int_{\theta_\mathrm{e}=0}^{\pi} \left(\frac{\partial^2 V_\mathrm{i}}{\partial r_\mathrm{e}^2}\right) r_\mathrm{e}^4 \rho_\mathrm{E}(r_\mathrm{e}; c, a_\mathrm{e}) \sin(\theta_\mathrm{e})\, \mathrm{d}\theta_\mathrm{e} \mathrm{d}r_\mathrm{e} + O\left[\left(\frac{r_\mathrm{e}}{|\mathbf{R}_I - \mathbf{R}|}\right)^3\right].$$

Analogously to before, the electron-ion contribution is derived to first order in (c/a_e) and second order in $(r_\mathrm{e}/|\mathbf{R}_I - \mathbf{R}|)$, which is the leading-order analytical expansion about the density tight-binding limit in this model.

D. Electron-electron contribution

Finally, we compute the repulsive electron-electron contribution to the potential. Again the displacement vector between the ions is aligned along the north pole. The valence electrons are parameterized in spherical polar coordinates around each atom, as depicted in Fig. S7c. The electron-electron contribution in this case may be written as

$$E^{\mathrm{BO}}_{\text{e-e}}(\mathbf{R}; c, a_\mathrm{e}) = \sum_I \iint V_\mathrm{e}(\mathbf{R}_I - \mathbf{R} + \mathbf{r}_\mathrm{e} - \mathbf{r}) \rho_\mathrm{E}(\mathbf{r}_\mathrm{e}; c, a_\mathrm{e}) \rho_\mathrm{E}(\mathbf{r}; c, a_\mathrm{e})\, \mathrm{d}\mathbf{r}_\mathrm{e} \mathrm{d}\mathbf{r}.$$

Due to the spherical symmetry of each electron cloud, this contribution reduces exactly to Coulomb repulsion, such that

$$E^{\mathrm{BO}}_{\text{e-e}}(\mathbf{R}; a_\mathrm{e}) = \frac{a_0^2}{a_\mathrm{e}^2} \sum_I \frac{1}{|\mathbf{R}_I - \mathbf{R}|}.$$

Note the total potential energy of the system at this stage, $E^{\mathrm{BO}}_{\text{i-i}} + E^{\mathrm{BO}}_{\text{e-i}} + E^{\mathrm{BO}}_{\text{e-e}}$, tends to zero as $(c/a_\mathrm{e}) \to 0$ and $|\mathbf{R}| \gg a_\mathrm{e}$. In this limit, the electrons are effectively on top of the ions and the whole system is neutral due to Gauss' theorem.

E. Pauli repulsion

To complement our result for the energy, we estimate the Pauli repulsion felt by the overlapping electron clouds. Since we only consider spherically symmetric (i.e. s-type) orbitals in the toy model, this reduces to a one-dimensional problem. We consider a Dirac delta potential well, of depth g, inside an infinite square well, such that:

$$V_{\text{well}}(x) = \begin{cases} -g\delta(x), & |x| = 0, \\ 0, & 0 < |x| < L, \\ \infty, & |x| \geq L. \end{cases}$$

In this scenario, g determines how tightly bound the electrons are to their respective atoms, and L represents the effective radius for the electron clouds. As L is reduced, the bound state energy is increased − this represents the energy increase due to the Pauli repulsion of overlapping orbitals.

The wavefunction takes the form $\Psi \propto \sinh(k(L - |x|))$ inside the infinite well, where k is the wave number. Considering the derivative continuity of the wavefunction at the origin, we derive the transcendental equation $\tanh y = \chi y$, where we have defined $y \equiv kL$ and $\chi \equiv \hbar^2/mgL$. We can derive an analytical form for the solution, and hence the scaling behavior of the energy with L, by finding the lowest root with a Newton-Raphson scheme. The iterative equation for the root is then

$$y_{n+1} = y_n - \frac{\tanh y_n - y_n\chi}{\text{sech}^2 y_n - \chi},$$

where $n \in \mathbb{Z}^+$. Since we are interested in the regime where the wavefunction is significantly influenced by the boundary wall, we take y_n to be small. Additionally, we are interested in the limit when Pauli repulsion is dominant i.e. when L is small. Taking these limits together, we find that $y_\infty = \sqrt{3\chi/2}$. Hence the energy of the bound state is

$$E^{\text{BO}}_{\text{Pauli}} = \frac{\hbar^2}{2mL^2} y_\infty^2 = \frac{3\hbar^2}{4m^{3/2}\sqrt{2E_{\text{b}}}L^3},$$

where we define the binding energy of an isolated Dirac delta potential well as $E_{\text{b}} \equiv mg^2/2\hbar^2$. In the density tight-binding approximation, the wavefunction takes the form $\Psi \propto \exp(-m^{1/2}\sqrt{2E_{\text{b}}}L/\hbar)$. Comparing this to the form of the wavefunction in Eq. S2 allows us to make the identification $E_{\text{b}} \sim a_{\text{e}}^{-2}$ up to physical constants. Hence, in atomic units, the energy gain due to Pauli repulsion becomes

$$E^{\text{BO}}_{\text{Pauli}} = \frac{3a_{\text{e}}}{4L^3}.$$

Note that due to the differences in unit cell geometry, the lattice constant cannot be directly compared between the various crystal structures. For this, we may examine the optimal effective radius of each atom in a spherical packing, defined as

$$r_{\text{eff}} = \left(\frac{3}{4\pi}\frac{V_{\text{u}}}{N_{\text{u}}}\right)^{1/3},$$

where V_{u} is the optimal volume of the unit cell, and N_{u} is the number of atoms enclosed. In place of L, we evaluate $E^{\text{BO}}_{\text{Pauli}}$ at the effective optimum radius. This rudimentary approximation for the Pauli repulsion allows us to analytically capture the scaling behavior as the lattice constant is reduced.

F. Crystal relaxation

Let us define the BO energy of the system as

$$E^{\text{BO}}(\mathbf{R}; c, a_{\text{e}}) = E^{\text{BO}}_{\text{i-i}}(\mathbf{R}; c, a_{\text{e}}) + E^{\text{BO}}_{\text{e-i}}(\mathbf{R}; c, a_{\text{e}}) + E^{\text{BO}}_{\text{e-e}}(\mathbf{R}; a_{\text{e}}) + E^{\text{BO}}_{\text{Pauli}}(a, a_{\text{e}}).$$

Note that there is an implicit lattice constant dependence in the first three terms in the form of the potentials, as well as in the lattice summations. Once we have calculated an analytical form for the BO energy of the system as a function of the displacement of the central atom, $\mathbf{R}$, and implicitly the lattice constant, a, we then compute the optimal lattice constant such that:

$$a_{\min} = \underset{a \in (a_{\text{e}}, \infty)}{\text{argmin}} \left(E^{\text{BO}}\right).$$

We subsequently relax the system to this lattice constant, compute the nuclear kinetic energy, and evaluate the total energy at a given $\mathbf{R}$. This renders the total energy, E, as a function of c and a_{e} only.

Crystal	$\rho_{\rm E}^{\rm osc}(\mathbf{r})/u$
cub/bcc/fcc	$\dfrac{1}{\tilde{N}_{\rm c.s.}}\sum_{i=1}^{\tilde{N}_{\rm c.s.}}\cos(\mathbf{r}\cdot\tilde{\mathbf{r}}_i^{\rm c.s.})$
dia	$\dfrac{1}{8}\left[\sum_{i=1}^{8}\cos(\mathbf{r}\cdot\tilde{\mathbf{r}}_i^{\rm dia})+\sum_{i=1}^{8}\cos\left(\left(\mathbf{r}-\mathbf{r}_{13}^{\rm dia}\right)\cdot\tilde{\mathbf{r}}_i^{\rm dia}\right)\right]$
hcp	$\dfrac{A_{\rm hcp}}{6}\left[\sum_{i=1}^{6}\cos(\mathbf{r}\cdot\tilde{\mathbf{r}}_i^{\rm hcp})\cos\left(\dfrac{3\pi}{\sqrt{6}a}z\right)+\sum_{i=1}^{6}\cos\left(\left(\mathbf{r}-\mathbf{r}_7^{\rm hcp}\right)\cdot\tilde{\mathbf{r}}_i^{\rm hcp}\right)\cos\left(\dfrac{3\pi}{\sqrt{6}a}\left(z-\dfrac{\sqrt{6}a}{3}\right)\right)\right]$
dhcp	$\dfrac{1}{6}\left[\dfrac{1}{3}\sum_{i=1}^{6}\cos(\mathbf{r}\cdot\tilde{\mathbf{r}}_i^{\rm dhcp})\cos\left(\dfrac{3\pi}{\sqrt{6}a}z\right)+\sum_{i=1}^{6}\cos\left(\left(\mathbf{r}-\mathbf{r}_7^{\rm dhcp}\right)\cdot\tilde{\mathbf{r}}_i^{\rm dhcp}\right)\cos\left(\dfrac{\sqrt{6}\pi}{a}\left(z-\dfrac{a}{\sqrt{6}}\right)\right)\right.$ $\left.+\dfrac{1}{3}\sum_{i=1}^{6}\cos\left(\left(\mathbf{r}-\mathbf{r}_{14}^{\rm dhcp}\right)\cdot\tilde{\mathbf{r}}_i^{\rm dhcp}\right)\cos\left(\dfrac{3\pi}{\sqrt{6}a}\left(z-\dfrac{\sqrt{6}a}{3}\right)\right)\right]$

TABLE S3. Oscillatory part of the electron cloud density in the density nearly-free electron model, $\rho_{\rm E}^{\rm osc}$, in units of the oscillation strength, u. $\tilde{N}_{\rm c.s.}$ is the number of displacement vectors, and $\{\tilde{\mathbf{r}}_i^{\rm c.s.}\}$ the set of displacements, in a unit cell of the reciprocal lattice. The vectors $\mathbf{r}_i^{\rm c.s.}$ are defined in Tables S1 and S2. The normalization constant, $A_{\rm hcp} = 2/3$, is chosen such that $\max(\rho_{\rm E}^{\rm osc}) = u$ for all crystal structures.

SVII. OSCILLATORY ELECTRON DENSITY IN THE DENSITY NEARLY-FREE ELECTRON MODEL

In order to approximate the oscillatory part of the electron cloud density in the density nearly-free electron model, we consider Fourier transforms of the reciprocal lattices, as shown in Table S3. For crystals with a single-ion basis, the resulting function has a simple form. However, for crystals with more than one ion in the basis, we consider a superposition of multiple offset lattices[S7]; with modulation along the z-axis, where appropriate. The functions, $\rho_{\rm E}^{\rm osc}$, are scaled such that $\max(\rho_{\rm E}^{\rm osc}) = u$ for all crystal structures. Over a unit cell, all of the functions integrate to zero.

[S1] E. Gkioulekas, Int. J. Sci. Math. Educ. **45**, 118 (2014).
[S2] M. A. H. Nerenberg, Int. J. Sci. Math. Educ. **22**, 303 (1991).
[S3] W. R. Inc., "Mathematica, Version 11.3," (2018).
[S4] P. P. Ewald, Ann. Phys. **369**, 253 (1921).
[S5] The $\sum'$ denotes that the $i = j$ term is omitted for $I = 0$.
[S6] P. Gibbon and G. Sutmann, Quantum Simulations of Complex Many-Body Systems: From Theory to Algorithms **10** (2002).
[S7] Note that the offsets given in Table S3 are not unique.
